# Efficacy and tolerability of artemisinin-based and quinine-based treatments for uncomplicated falciparum malaria in pregnancy: a systematic review and individual patient data meta-analysis

**DOI:** 10.1016/S1473-3099(20)30064-5

**Published:** 2020-08

**Authors:** Makoto Saito, Rashid Mansoor, Kalynn Kennon, Anupkumar R Anvikar, Elizabeth A Ashley, Daniel Chandramohan, Lauren M Cohee, Umberto D'Alessandro, Blaise Genton, Mary Ellen Gilder, Elizabeth Juma, Linda Kalilani-Phiri, Irene Kuepfer, Miriam K Laufer, Khin Maung Lwin, Steven R Meshnick, Dominic Mosha, Victor Mwapasa, Norah Mwebaza, Michael Nambozi, Jean-Louis A Ndiaye, François Nosten, Myaing Nyunt, Bernhards Ogutu, Sunil Parikh, Moo Kho Paw, Aung Pyae Phyo, Mupawjay Pimanpanarak, Patrice Piola, Marcus J Rijken, Kanlaya Sriprawat, Harry K Tagbor, Joel Tarning, Halidou Tinto, Innocent Valéa, Neena Valecha, Nicholas J White, Jacher Wiladphaingern, Kasia Stepniewska, Rose McGready, Philippe J Guérin

**Affiliations:** a WorldWide Antimalarial Resistance Network (WWARN), Oxford, UK; b Infectious Diseases Data Observatory (IDDO), Oxford, UK; c Nuffield Department of Medicine, Centre for Tropical Medicine and Global Health, University of Oxford, Oxford, UK; d Indian Council of Medical Research, National Institute of Malaria Research, New Delhi, India; e Lao-Oxford-Mahosot Hospital-Wellcome Trust Research Unit, Vientiane, Laos; f Department of Disease Control, London School of Hygiene and Tropical Medicine, London, UK; g Center for Vaccine Development and Global Health, University of Maryland School of Medicine, Baltimore, MD, USA; h Medical Research Council Unit, The Gambia at the London School of Hygiene & Tropical Medicine, Banjul, The Gambia; i Department of Epidemiology and Public Health, Swiss Tropical and Public Health Institute, University of Basel, Basel, Switzerland; j University Center of General Medicine and Public Health, Lausanne, Switzerland; k Shoklo Malaria Research Unit, Mahidol-Oxford Tropical Medicine Research Unit, Faculty of Tropical Medicine, Mahidol University, Bangkok, Thailand; l Kenya Medical Research Institute, Nairobi, Kenya; m Department of Medicine, University of Malawi College of Medicine, Blantyre, Malawi; n Department of Epidemiology, Gillings School of Global Public Health, University of North Carolina, NC, USA; o Ifakara Health Institute, Dar es Salaam, Tanzania; p Infectious Disease Research Collaboration, Makerere University, Kampala, Uganda; q Department of Clinical Sciences, Tropical Diseases Research Centre, Ndola, Zambia; r Department of Parasitology, Université Cheikh Anta Diop, Dakar, Senegal; s Duke Global Health Institute, Duke University, Durham, NC, USA; t Department of Epidemiology of Microbial Diseases, Yale School of Public Health, New Haven, CT, USA; u Myanmar–Oxford Clinical Research Unit, Yangon, Myanmar; v Epidemiology and Public Health Unit, Institut Pasteur du Cambodge, Phnom Penh, Cambodia; w Department of Obstetrics and Gynecology, Division of Woman and Baby, University Medical Center Utrecht, Utrecht, Netherlands; x School of Medicine, University of Health and Allied Sciences, Ho, Ghana; y Clinical Research Unit of Nanoro, Institut de Recherche en Sciences de la Santé, Nanoro, Burkina Faso

## Abstract

**Background:**

Malaria in pregnancy affects both the mother and the fetus. However, evidence supporting treatment guidelines for uncomplicated (including asymptomatic) falciparum malaria in pregnant women is scarce and assessed in varied ways. We did a systematic literature review and individual patient data (IPD) meta-analysis to compare the efficacy and tolerability of different artemisinin-based or quinine-based treatments for malaria in pregnant women.

**Methods:**

We did a systematic review of interventional or observational cohort studies assessing the efficacy of artemisinin-based or quinine-based treatments in pregnancy. Seven databases (MEDLINE, Embase, Global Health, Cochrane Library, Scopus, Web of Science, and Literatura Latino Americana em Ciencias da Saude) and two clinical trial registries (International Clinical Trials Registry Platform and ClinicalTrials.gov) were searched. The final search was done on April 26, 2019. Studies that assessed PCR-corrected treatment efficacy in pregnancy with follow-up of 28 days or more were included. Investigators of identified studies were invited to share data from individual patients. The outcomes assessed included PCR-corrected efficacy, PCR-uncorrected efficacy, parasite clearance, fever clearance, gametocyte development, and acute adverse events. One-stage IPD meta-analysis using Cox and logistic regression with random-effects was done to estimate the risk factors associated with PCR-corrected treatment failure, using artemether-lumefantrine as the reference. This study is registered with PROSPERO, CRD42018104013.

**Findings:**

Of the 30 studies assessed, 19 were included, representing 92% of patients in the literature (4968 of 5360 episodes). Risk of PCR-corrected treatment failure was higher for the quinine monotherapy (n=244, adjusted hazard ratio [aHR] 6·11, 95% CI 2·57–14·54, p<0·0001) but lower for artesunate-amodiaquine (n=840, 0·27, 95% 0·14–0·52, p<0·0001), artesunate-mefloquine (n=1028, 0·56, 95% 0·34–0·94, p=0·03), and dihydroartemisinin-piperaquine (n=872, 0·35, 95% CI 0·18–0·68, p=0·002) than artemether-lumefantrine (n=1278) after adjustment for baseline asexual parasitaemia and parity. The risk of gametocyte carriage on day 7 was higher after quinine-based therapy than artemisinin-based treatment (adjusted odds ratio [OR] 7·38, 95% CI 2·29–23·82).

**Interpretation:**

Efficacy and tolerability of artemisinin-based combination therapies (ACTs) in pregnant women are better than quinine. The lower efficacy of artemether-lumefantrine compared with other ACTs might require dose optimisation.

**Funding:**

The Bill & Melinda Gates Foundation, ExxonMobil Foundation, and the University of Oxford Clarendon Fund.

## Introduction

The physiological changes that occur during pregnancy mean that pregnant women are more susceptible to malaria than women who are not pregnant. Symptomatic and asymptomatic infections affect both mother and fetus.^1^, ^2^ Around 60% of pregnant women in the world live in malaria-endemic regions, with an estimated 125 million pregnant women at risk of malaria every year.^3^ Efficacious prevention and treatment are required to limit maternal mortality and the cumulative adverse effects of malaria episodes during pregnancy. However, this susceptible population nurturing future generations is understudied.^4^ Pregnant women are often excluded from clinical trials and antimalarial studies are no exception.^5^

Over the past 30 years, pregnant women have been systematically excluded from standard antimalarial treatment efficacy studies.^6^ There were several reasons for this exclusion, the first of which was concern over the teratogenicity and embryotoxicity of artemisinin derivatives shown in animal studies.^7^ Since the mid-1990s, however, data for the human safety of artemisinin derivatives in the first trimester has been accumulating, leading to the conclusion that artemisinin derivatives are at least as safe as quinine.^8^, ^9^ The second reason was that there are no agreed guidelines to assess antimalarial drug efficacy during pregnancy. The 2009 WHO guidelines^10^ on the assessment of antimalarial efficacy recommend the exclusion of pregnant women from treatment efficacy studies on the basis that they are different from the non-pregnant population in several ways, including altered immunity, gestational physiological changes affecting pharmacokinetics and pharmacodynamics, and the presence of the placenta, which might provide a haven for malaria parasites.^11^, ^12^, ^13^

**Research in context**

**Evidence before this study**

We did a systematic literature search for studies assessing treatment efficacy of artemisinin-based or quinine-based treatments for uncomplicated falciparum malaria (including asymptomatic malaria) in pregnancy using seven databases (MEDLINE, Embase, Global Health, Cochrane Library, Scopus, Web of Science, and Literatura Latino Americana em Ciências da Saúde) and two clinical trial registries (International Clinical Trials Registry Platform and ClinicalTrials.gov). The final search was done on April 26, 2019, without restrictions on publication year or language. Previous aggregated data meta-analyses showed a lower efficacy of quinine-based treatment than artemisinin-based treatments, though the strength of evidence was low as the number of randomised control trials (RCTs) was very small, and different study designs and outcome measures were used. It was impossible to compare efficacy of different ACTs because of the paucity of RCTs.

**Added value of this study**

This meta-analysis includes the largest dataset of malaria treatments in pregnancy, being composed of individual patient data of 4968 episodes in 19 studies across ten countries, representing 92% (4968 of 5360 episodes) of data identified in the systematic literature review. Quinine is recommended to treat malaria in the first trimester of pregnancy and is still commonly used for all trimesters. Our study shows that quinine was less preferable than ACTs because of higher treatment failure, unless combined with clindamycin, higher occurrence of acute adverse events (ie, lower tolerability), and higher risk of gametocyte development (ie, higher risk of transmission). Artemether-lumefantrine (AL), the most commonly used ACT, shows the best tolerability but a lower efficacy than other standard ACTs. In moderate-to-high falciparum malaria transmission areas, the risk of treatment failure was higher in nulliparous women. Dose optimisation of AL for pregnant women should be further investigated. Although the numbers of first trimester pregnancies studied were small, these findings support recommendations in 2017 that ACTs should replace quinine as the treatment of choice for falciparum malaria in all trimesters.

**Implications of all the available evidence**

This meta-analysis, together with previous research on the safety of ACTs in the first trimester, provides compelling evidence that both efficacy and safety of ACTs in pregnant women are better than quinine-based treatments. Suboptimal dosing of lumefantrine with pregnant women might explain a slightly lower efficacy of AL than other ACTs. Dose optimisation of antimalarial drugs in pregnancy, supported by pharmacokinetic studies, will be required to achieve the highest treatment success in pregnancy to protect both mother and fetus from the adverse effects of malaria infection.

Considering the variability of study designs and the fact that most antimalarial studies in pregnancy were single-arm studies, conclusions from conventional aggregated meta-analyses were not reliable.^6^ There is little evidence to support the WHO recommendation^14^ of quinine (with clindamycin if available) for women in their first trimester and artemisinin-based combination therapy (ACT) for women in their second or third trimester.

By including single-arm studies and more studies done after the release of the treatment guidelines, this study aims to contribute to the body of evidence by pooling individual patient data from studies to assess the efficacy of recommended antimalarial drugs for uncomplicated falciparum malaria during pregnancy, including patients who were asymptomatic. Our objective was to compare the efficacy and tolerability of artemisinin-based treatments (ABT) and quinine-based treatments (QBT), and also assess the difference between ACTs using artemether-lumefantrine (AL), the most used ACT, as the reference standard.

## Methods

### Search strategy and selection criteria

We did a systematic review on the efficacy of ABT and QBT on uncomplicated falciparum malaria, including asymptomatic parasitaemia (hereafter referred as uncomplicated falciparum malaria) in pregnancy without any restrictions on language or publication year, and this was published elsewhere.^6^ Two reviewers (MS and MEG) assessed eligibility independently, and discrepant results were resolved by a second assessment. Seven databases (MEDLINE, Embase, Global Health, Cochrane Library, Scopus, Web of Science, and LILACS) and two clinical trial registries (ICTRP and ClinicalTrial.gov) were used. Prospective treatment efficacy studies of uncomplicated falciparum malaria, including pregnant women in any trimester, were identified using a combination of five components: malaria, pregnancy, treatment or names of antimalarial drugs, study design (interventional or observational cohort studies), and outcome types (efficacy), up until the final date of April 26, 2019.

Studies were included in this meta-analysis if *Plasmodium falciparum* parasitaemia was confirmed by microscopy before treatment, regardless of the patient's symptoms, the length of active follow-up was 28 days and over, and PCR was used to differentiate the recurrence of infections with *P falciparum* during follow-up.^10^ Investigators of published and unpublished studies that were identified by the systematic literature review were invited to share the individual patient data with the WorldWide Antimalarial Resistance Network. The protocol of the methods used to standardise the individual patient data and statistical analyses was published elsewhere,^15^ and the study is registered on PROSPERO (CRD42018104013).

### Outcomes

Pregnancy outcomes are reported elsewhere.^16^ Only patients who were treated with a full course of antimalarial drugs of recommended regimen according to the WHO treatment guidelines were included in the analyses.^14^ Patients with indeterminate PCR were excluded from PCR-corrected efficacy analyses in the main analysis,^10^ but we also did sensitivity analyses assuming that the patients were either recrudescent or reinfected. Fever clearance was defined as the absence of fever (>37·5°C) and parasite clearance as the absence of microscopic asexual parasitaemia on days 1, 2, or 3. Gametocyte positivity on day 7 was assessed by only including patients who did not have gametocytaemia on day 0 and who did not have recurrence of *P falciparum* within 28 (±3) days. Acute adverse events were regarded as present if the symptom was actively assessed and recorded on any day between days 1 and 7.

### Statistical analysis

All individual patient data from each study were pooled together for statistical analyses. For the descriptive analysis, treatment efficacy, and gametocyte positivity for each treatment at fixed timepoints (ie, days 28, 42, and 63 for efficacy, and day 7 for gametocyte positivity) were estimated by the Kaplan-Meier method in each study at each site. Then, after complementary log-log transformation,^17^ these estimates were pooled by the aggregated data meta-analysis approach (a two-stage IPD meta-analysis).

We did a one-stage IPD meta-analysis using the Cox proportional hazards regression with shared frailty for study sites to identify the risk factors for recrudescence and to compare different treatment drugs. We used univariable and multivariable mixed effects logistic regression models to model risk of parasite positivity, gametocyte positivity, and acute adverse events. We used hazard ratios (HRs) for PCR-corrected efficacy and ORs for the other outcomes.

We did a complete case analysis because of the small proportion of missing data for the main variables of interest. For all regression models, we identified independent risk factors by backward elimination.^15^ Two variables, treatment regimen and baseline asexual parasitaemia, were included in the multivariable models on PCR-corrected efficacy as a priori forced variables, regardless of statistical significance (p<0·05). Few women had multiple episodes recorded, thus previous history of malaria 28 days before treatment was included taking account for this within-person correlation. Interaction between parity and malaria transmission intensity (categorised into three groups as defined in the protocol),^15^ or age and transmission was assessed if age or parity was included in the multivariable model, as the effect of age and parity (ie, pregnancy-specific immunity) can be different depending on transmission intensity. Any antimalarial use (including intermittent preventive treatment) was censored on the documented day. *Plasmodium vivax* intercalated infection (ie, *P vivax* mono-infection before the recurrence of *P falciparum* parasitaemia) was regarded as a time-dependent covariate if the original study genotyped falciparum recurrences regardless of intercalated infection with *P vivax*. Otherwise, intercalated infection with *P vivax* was regarded as censored following the WHO guidelines.^10^ AL, the most commonly used drug, was used as the reference group, and comparison between every pair of drugs was not done to avoid multiple testing. In the Cox regression, global test was used for checking the proportional hazard assumption. We used the piecewise-Cox model by introducing time-by-covariate interactions if the proportional-hazards assumption of constant HRs was violated. As a prespecified sensitivity analysis, the model was refitted, excluding one study site at a time to identify any influential studies. For the other outcomes, the treatment group was always included in the final models. Risk of bias was assessed using the Cochrane risk of bias assessment tool for randomised controlled trials (RCTs) and the Newcastle-Ottawa scale for observational cohort studies.

Statistical analysis was done using R (R Foundation for Statistical Computing, Vienna, Austria) or Stata MP15.1 (StataCorp, College Station, TX, USA).

### Role of the funding source

The funders did not participate in the study design, the writing of the paper, decision to publish, or preparation of the manuscript.

## Results

**Figure 1:**
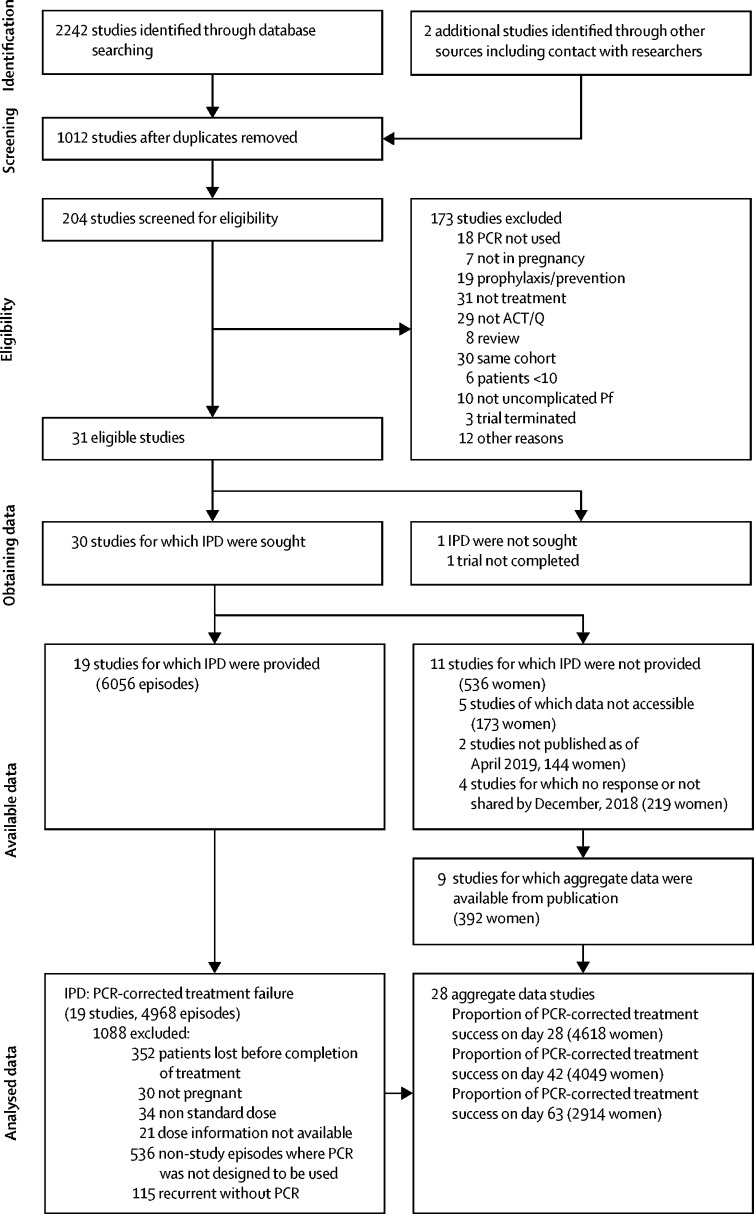
Study selection ACT=artemisinin-based combination therapies. IPD=individual patient data. Q=quinine monotherapy.

Our search identified 30 studies with PCR-corrected efficacy. With the exception of two unpublished studies, individual patient data from 4968 (92%) of 5360 episodes in 19 studies^18^, ^19^, ^20^, ^21^, ^22^, ^23^, ^24^, ^25^, ^26^, ^27^, ^28^, ^29^, ^30^, ^31^, ^32^, ^33^, ^34^, ^35^, ^36^ were shared and pooled for the analyses of PCR-corrected efficacy (figure 1).

The included studies were done between 1995 and 2014 in ten different countries (appendix pp 2–4): nine studies (comprising 3813 episodes) were done in sub-Saharan Africa and ten (comprising 1155 episodes) in Asia.

Ten antimalarial treatments were included in this analysis: AL (1278 [89·7%] of 1425 episodes in the literature), artesunate-amodiaquine (ASAQ; 840 [91·8%] of 915 episodes), artesunate-mefloquine (ASMQ; 1028 [96·0%] of 1071 episodes), dihydroartemisinin-piperaquine (DP; 872 [98·6%] of 884 episodes), artesunate-sulfadoxine-pyrimethamine (ASSP; 173 [84·4%] of 205 episodes), artesunate monotherapy (230 [100%] of 230 episodes), artesunate with clindamycin (AC; 142 [100%] of 142 episodes), artesunate-atovaquone-proguanil (AAP; 91 [100%] of 91 episodes), quinine monotherapy (244 [81·6%] of 299 episodes), and quinine with clindamycin (QC; 67 [100%] of 67 episodes). The fixed dose formulation was used in all participants who took ASAQ and in 962 (93·6%) of the 1028 participants who took ASMQ (appendix pp 5–7).

Of the 4968 episodes in 4745 women, the mean age was 23·5 years (SD 6·0), and the median parity was one (range zero to ten; table 1). Most episodes were either in the second trimester (weeks 14–27, 3325 [67·0%] of 4965 episodes) or third trimester (≥28 weeks, 1610 [32·4%] of 4965 episodes) with 33 (0·7%) of 4968 episodes in the first trimester (<14 weeks). Of these, 4914 (98·9%) of 4968 episodes were *P falciparum* mono-infections. The geometric mean parasite density before treatment was 1189 (range 1–447 638) asexual parasites per μL, with 79 (1·6%) of 4968 episodes considered to be hyperparasitaemic (defined as >100 000 parasites per μL). Only 504 (10·4%) of the 4854 women were febrile (>37·5°C) at presentation.

**Table 1:** Baseline characteristics of the pregnant women assessed for PCR-corrected treatment efficacy

		**AL**	**AAP**	**AC**	**AS**	**ASAQ**	**ASMQ**	**ASSP**	**DP**	**Q**	**QC**
Age (years)		21 (14–45)	23 (16–38)	25 (15–43)	25·5 (14–44)	22 (15–46)	22 (15–45)	22 (15–42)	21 (15–43)	22 (16–41)	25 (15–41)
Parity		1 (0–10)	1 (0–8)	1 (0–9)	2 (0–9)	1 (0–8)	1 (0–10)	0 (0–5)	1 (0–9)	1 (0–8)	1 (0–7)
Gravidity		2 (1–13)	2 (1–9)	3 (1–13)	3 (1–11)	2 (1–10)	2 (1–13)	2 (1–8)	2 (1–12)	2 (1–12)	3 (1–9)
Height (cm)		156·0 (130–184)	153·0 (124–161)	152·0 (140–166)	151·0 (140–167)	158·0 (132–179)	156·0 (125–187)	149·8 (134–186)	155·0 (125–178)	153·0 (141–166)	154·0
1st trimester*		2/1277 (0·2%)	2/91 (2·2%)	0/142	5/230 (2·2%)	0/840	3/1028 (0·3%)	1/172 (0·6%)	7/872 (0·8%)	6/243 (2·5%)	1/67 (1·5%)
2nd trimester*		876/1277 (69%)	53/91 (58%)	54/142 (38%)	122/230 (53%)	624/840 (74%)	666/1028 (65%)	122/172 (71%)	592/872 (68%)	176/243 (72%)	40/67 (60%)
3rd trimester*		399/1277 (31%)	36/91 (40%)	88/142 (62%)	103/230 (45%)	216/840 (26%)	359/1028 (35%)	49/172 (29%)	273/872 (31%)	61/243 (25%)	26/67 (39%)
Haemoglobin (g/dL)		10·2 (4·1–15·0)	9·3 (6·2–12·5)	9·4 (5·5–13·9)	9·4 (5·1–13·4)	10·1 (5·3–15·0)	9·9 (4·6–14·3)	9·2 (7·0–14·0)	10·0 (4·8–15·0)	10·1 (5·9–14·4)	9·3 (3·6–12·6)
Weight (kg)		53 (34–88)	49 (39–69)	51 (38–67)	49 (37–74)	55 (40–107)	53 (33–98)	48 (31–65)	54 (35–115)	52 (30–98)	49 (40–64)
Fever*		119/1232 (9·7%)	23/91 (25·3%)	26/133 (19·5%)	55/228 (24·1%)	52/841 (6·2%)	97/1027 (9·4%)	31/121 (25·6%)	42/871 (4·8%)	46/243 (18·9%)	13/67 (19·4%)
Parasitaemia (log_10_/μL)		3·1 (0–5·7)	3·6 (1·5–5·5)	3·3 (1·2–5·5)	3·4 (1·2–5·5)	2·8 (1·2–5·0)	3·0 (1·2–5·4)	3·3 (1·5–5·3)	2·9 (0·7–5·6)	3·3 (1·6–5·3)	3·3 (1·2–5·0)
Gametocytaemia*		54/1242 (4·3%)	4/91 (4·4%)	18/141 (12·8%)	21/225 (9·3%)	24/815 (2·9%)	17/1007 (1·7%)	4/122 (3·3%)	32/874 (3·7%)	18/243 (7·4%)	5/66 (7·6%)
Pf mono-infection*		1265/1278 (99·0%)	90/91 (98·9%)	133/142 (93·7%)	214/230 (93·0%)	841/841 (100·0%)	1028/1028 (100·0%)	173/173 (100·0%)	864/874 (98·9%)	243/244 (99·6%)	63/67 (94·0%)
Malaria transmission*											
	Low	200/1278 (15·6%)	91/91 (100·0%)	141/142 (99·3%)	230/230 (100·0%)	26/841 (3·1%)	188/1028 (18·3%)	122/173 (70·5%)	82/874 (9·4%)	172/244 (70·5%)	67/67 (100·0%)
	Moderate	776/1278 (60·7%)	0	1/142 (0·7%)	0	482/841 (57·3%)	507/1028 (49·3%)	18/173 (10·4%)	738/874 (84·4%)	72/244 (29·5%)	0
	High	302/1278 (23·6%)	0	0	0	333/841 (39·6%)	333/1028 (32·4%)	33/173 (19·1%)	54/874 (6·2%)	0	0

Fever is defined as body temperature >37·5°C. Trimesters are categorised as follows: first (≤13 weeks), second (14–27 weeks), and third (≥28 weeks). AAP=artesunate with atovaquone-proguanil. AC=artesunate with clindamycin. AL=artemether-lumefantrine. AS=artesunate monotherapy. ASAQ=artesunate-amodiaquine. ASMQ=artesunate-mefloquine. ASSP=artesunate-sulfadoxine-pyrimethamine. DP=dihydroartemisinin-piperaquine. Pf=*Plasmodium falciparum*. Q=quinine monotherapy. QC=quinine with clindamycin.

* Data are presented as the number in the category divided by total number assessed.

For all patients who took ABTs or QC, PCR-corrected treatment efficacy by the Kaplan-Meier method was more than 90% at day 28, 42, or 63, pooled by random effects (table 2, and appendix p 8). Quinine monotherapy had the lowest pooled efficacy at 87·7 % (95% CI 58·2–99·3%, n=181 in three studies) on day 28.

**Table 2:** PCR-corrected treatment efficacy for each treatment at fixed timepoints in each shared study pooled by random effects

	**Day 28**		**Day 42**		**Day 63**	
	N	% (95% CI)	N	% (95% CI)	N	% (95% CI)
AL	1168	96·9 (94·5–98·5)	929	95·5 (92·6–97·5)	598	93·6 (89·1–96·7)
AAP	58	99·9 (98·4–100·0)	49	98·9 (91·5–100·0)	40	98·5 (91·4–99·9)
AC	106	99·5 (97·4–99·9)	73	98·6 (85·4–100·0)	39	97·5 (71·4–100·0)
AS	169	95·9 (88·4–99·1)	140	95·6 (87·3–99·1)	91	95·3 (85·7–99·2)
ASAQ	811	99·6 (99·2–99·9)	782	99·2 (98·5–99·7)	611	98·8 (97·2–99·6)
ASMQ	982	99·9 (99·6–100·0)	948	99·5 (98·3–99·9)	717	99·2 (97·0–99·9)
ASSP	159	99·0 (95·4–99·9)	120	99·2 (95·7–99·9)	113	99·2 (95·7–99·9)
DP	815	99·5 (98·0–99·9)	799	99·4 (97·0–99·9)	705	98·4 (95·6–99·6)
Q	181	87·7 (58·2–99·3)	164	86·8 (56·6–99·3)	128	84·9 (51·6–99·3)
QC	43	99·9 (97·6–100·0)	37	99·9 (97·0–100·0)	28	99·9 (96·3–100·0)

Treatment success was estimated by pooling the Kaplan-Meier survival estimates in each study by random effects method. AAP=artesunate with atovaquone-proguanil. AC=artesunate with clindamycin. AL=artemether-lumefantrine. AS=artesunate monotherapy. ASAQ=artesunate-amodiaquine. ASMQ=artesunate-mefloquine. ASSP=artesunate-sulfadoxine-pyrimethamine. DP=dihydroartemisinin-piperaquine. Q=quinine monotherapy. QC=quinine with clindamycin.

In univariable analysis, four risk factors were associated with recrudescence: treatment, age, baseline asexual parasite density, and parity (table 3). After adjustment for other risk factors in the multivariable analysis, the risk of PCR-corrected treatment failure compared with AL was decreased for ASAQ (aHR 0·27, 95% CI 0·14–0·52, p<0·0001), ASMQ (0·56, 0·34–0·94, p=0·03), DP (0·35, 0·18–0·68, p=0·002), and AC (0·37, 0·15–0·91, p=0·03), but higher in quinine monotherapy (6·11, 2·57–14·54, p<0·0001). For AAP, artesunate monotherapy, ASSP, and QC, the risk of failure was not statistically different to that for AL (table 3). Higher baseline parasite density was associated with an increased risk of treatment failure (1·93 per ten-fold increase, 1·61–2·32, p<0·0001).

**Table 3:** Risk factors for PCR-corrected treatment failure in pregnant women infected with falciparum malaria

		**Number assessed (failure)**	**Univariable analysis**		Multivariable analysis*	
			HR (95% CI)	p-value	HR (95% CI)	p-value
**Treatment**						
	AL	1278 (68)	..	..	..	..
	AAP	91 (2)	0·39 (0·07–2·11)	0·27	0·31 (0·06–1·61)	0·17
	AC	142 (6)	0·46 (0·19–1·15)	0·10	0·37 (0·15–0·91)	0·03
	AS	230 (15)	0·72 (0·37–1·42)	0·35	0·64 (0·34–1·23)	0·18
	ASAQ	841 (12)	0·27 (0·14–0·52)	<0·0001	0·27 (0·14–0·52)	<0·0001
	ASMQ	1028 (25)	0·56 (0·33–0·94)	0·03	0·56 (0·34–0·94)	0·03
	ASSP	173 (4)	1·68 (0·30–9·31)	0·55	2·05 (0·38–11·03)	0·40
	DP	874 (14)	0·38 (0·20–0·74)	0·004	0·35 (0·18–0·68)	0·002
	Q	244 (31)	5·70 (2·09–15·55)	<0·0001	6·11 (2·57–14·54)	<0·0001
	QC	67 (1)	0·63 (0·05–7·22)	0·71	0·48 (0·04–5·24)	0·55
**Age (years)**		4968 (178)	0·96 (0·93–0·98)	0·002	..	..
Parity						
	0	2130 (89)	..	..	..	..
	1	1040 (31)	0·60 (0·40–0·90)	0·01	0·59 (0·39–0·89)	0·01
	≥2	1733 (56)	0·51 (0·36–0·72)	0·0002	0·62 (0·44–0·89)	0·009
**Trimester**						
	1	27 (1)	0·45 (0·06–3·31)	0·44	..	..
	2	3325 (131)	..	..	..	..
	3	1610 (46)	0·81 (0·58–1·15)	0·24	..	..
Weight (kg)		4943 (177)	1·00 (0·98–1·03)	0·78	..	..
Parasitaemia (log_10_/μL)		4968 (178)	1·97 (1·65–2·35)	0·0001	1·93 (1·61–2·32)	<0·0001
**Hyperparasitaemia**						
	Yes	79 (6)	1·74 (0·76–4·00)	0·19	..	..
	No	4889 (172)	..	..	..	..
**Fever >37·5°C**						
	Yes	504 (32)	1·46 (0·98–2·18)	0·07	..	..
	No	4350 (142)	..	..	..	..
Haemoglobin (g/dL)		4917 (175)	0·97 (0·88–1·08)	0·60	..	..
**Presence of gametocytes**						
	Yes	197 (12)	1·28 (0·70–2·33)	0·42	..	..
	No	4629 (157)	..	..	..	..
**Mixed infection**						
	Yes	54 (5)	1·76 (0·70–4·43)	0·23	..	..
	No	4914 (173)	..	..	..	..
**Intercalated vivax infection**						
	Yes	233 (10)	0·59 (0·30–1·18)	0·14	..	..
	No	4735 (168)	..	..	..	..
**Transmission intensity**						
	Low	1319 (81)	1·75 (0·46–6·73)	0·41	..	..
	Moderate	2594 (49)	1·26 (0·30–5·26)	0·75	..	..
	High	1055 (48)	..	..	..	..

p value for shared frailty <0·001. AAP=artesunate with atovaquone-proguanil. AC=artesunate with clindamycin. AL=artemether-lumefantrine. AS=artesunate monotherapy. ASAQ=artesunate-amodiaquine. ASMQ=artesunate-mefloquine. ASSP=artesunate-sulfadoxine-pyrimethamine. DP=dihydroartemisinin-piperaquine. HR=hazard ratio. Q=quinine monotherapy. QC=quinine with clindamycin.

* Adjusted by treatment, parity, parasitaemia, and previous antimalarial treatment.

The effect of parity was different depending on the malaria transmission intensity (p value for interaction 0·02). Compared with nulliparous women, the risks of treatment failure were decreased in primiparous women in moderate (aHR 0·29, 95% CI 0·10–0·81, p=0·02) and high transmission areas (0·73, 0·34–1·57, p=0·42); and in multiparous women in moderate (0·22, 0·08–0·63, p=0·004) and high transmission areas (0·49, 0·25–0·95, p=0·04); but not different in low transmission areas.

Sensitivity analyses were done by a piece-wise Cox model that split the observation time to satisfy the proportionality assumption and also for different handling of indeterminate PCR and *P vivax* intercalated infection. The estimates for these models were similar to the main analysis above (appendix pp 13–16).

Parasite clearance on days 1 and 2 was analysed, including those with recurrent infection that was not genotyped, or was indeterminate (appendix pp 19–22). The adjusted risk of positive parasitaemia by microscopy on day 2 (n=4876) was higher for quinine monotherapy (adjusted OR [aOR] 33·64, 95% CI 13·63–83·00, p<0·0001) and QC (22·92, 7·90–66·50, p<0·0001) than for AL. The risk was not different in ACTs compared with AL. Fever clearance is described in the appendix (p 23).

The number of patients with gametocytaemia on different days, stratified by baseline gametocytaemia, is summarised in the appendix (p 9). Further analyses of the risk of gametocyte carriage in women without gametocytaemia on day 0 were done by categorising treatments into ABT and QBT because of the small number of outcomes in each treatment. Overall, the pooled Kaplan-Meier estimates of women without gametocytes at baseline but positive by day 7 were 4·0% (95% CI 2·3–6·5, n=4256 in 18 studies) after ABT and 23·9% (7·0–46·3, n=261 in five studies) after QBT.

There were 3445 women who were without gametocytaemia on day 0 and were assessed for the risk of gametocytaemia on day 7. In the univariable analysis, the risk of positive gametocytaemia on day 7 was higher after QBT than after ABT (OR 8·50, 95% CI 2·55–28·33, p=0·0005; appendix p 10). In the multivariable analyses adjusted for other risk factors, the risk of developing gametocytaemia was still higher after QBT than after ABT (aOR 7·38, 2·29–23·82, p=0·001). Higher baseline parasitaemia (aOR 1·82 per ten-fold increase, 1·23–2·71, p=0·003) and lower bodyweight (aOR 0·93 per kg, 0·87–0·99, p=0·02) were associated with an increased risk of developing gametocytaemia. Compared with women who were nulliparous, the risks of developing gametocytaemia were decreased in women who were primiparous (aOR 0·41, 0·15–1·10, p=0·08) or multiparous (aOR 0·38, 0·17–0·89, p=0·03). Low malaria transmission intensity was associated with a higher adjusted risk of developing gametocytaemia (aOR 8·12, 1·39–47·55, p=0·02) than moderate transmission intensity areas.

The PCR-uncorrected treatment efficacy on days 28, 42, or 63 by the Kaplan-Meier method was pooled by random effects for each malaria transmission intensity (appendix pp 11–12). In high transmission areas, PCR-uncorrected treatment efficacy of quinine on day 28 was 42·0% (95% CI 27·7–59·8, n=64 in two studies) and AL was 86·2% (82·1–89·8, n=308 in three studies). In low transmission areas, efficacy was higher for both drugs: PCR-uncorrected efficacy of quinine on day 28 was 66·4% (42·2–88·6, n=128 in four studies) and AL was 92·7% (63·2–99·9, n=174 in two studies). Efficacy on day 28 was greater than 95% for all other ACTs regardless of transmission intensity.

**Figure 2:**
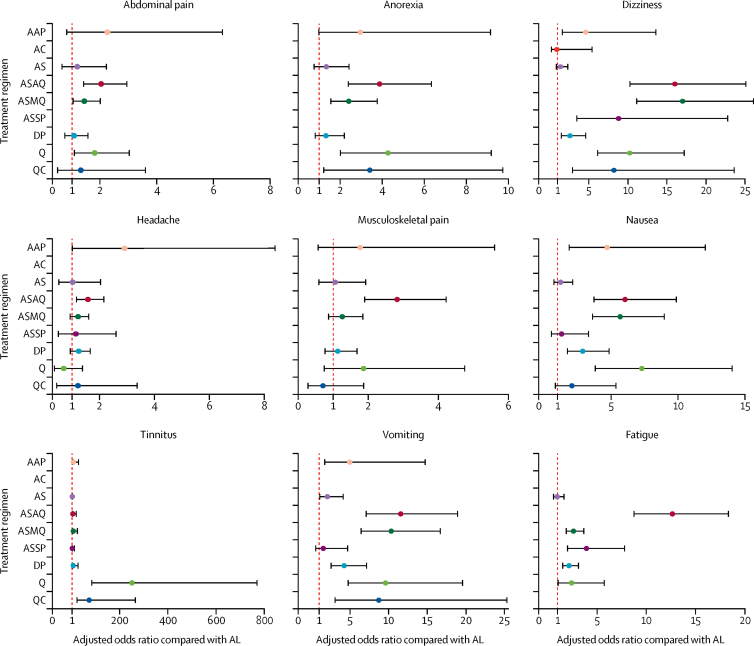
Adjusted odds ratio of developing symptoms after treatment compared with artemether-lumefantrine AAP=artesunate-atovaquone-proguanil. AC=artesunate with clindamycin. AL=artemether-lumefrantrine. AS=artesunate monotherapy. ASAQ=artesunate-amodiaquine. ASMQ=artesunate-mefloquine. ASSP=artesunate-sulfadoxine-pyrimethamine. DP=dihydroartemisinin-piperaquine. Q=quinine monotherapy. QC=quinine with clindamycin.

The risk of developing adverse events in the first week after treatment was estimated for nine commonly assessed symptoms (figure 2, appendix pp 24–36). Overall, the AL treatment group had the lowest risk, similar to artesunate monotherapy, followed by the DP treatment group. QBT, ASAQ, and ASMQ were associated with higher adjusted risks of abdominal pain, anorexia, dizziness, and vomiting than for AL. The risk of tinnitus was higher for quinine (aOR 249·84, 80·90–771·56, p<0·0001) and QC (71·91, 19·45–265·86, p<0·0001) than AL. The risks of musculoskeletal pain (2·83, 1·88–4·24, p<0·0001) and fatigue (12·65, 8·70–18·38, p<0·0001) were higher after ASAQ than after AL.

## Discussion

We present here the results of, to our knowledge, the largest IPD meta-analysis to date assessing the efficacy and tolerability of ABTs and QBTs for uncomplicated falciparum malaria in pregnancy, representing 92% of all patients enrolled in published studies. The PCR-corrected efficacy of evaluated antimalarial drugs was greater than 90% after 28–63 days of follow-up, except for quinine monotherapy, which is the recommended treatment for pregnant women with *P falciparum* infection in their first trimester. The slightly lower efficacy of AL, the most widely used ACT in pregnancy, compared with other standard ACTs (ie, ASAQ, ASMQ, and DP) in the multivariable analysis might be due to its under-dosing in pregnant women, particularly if the immunity level is low (appendix pp 17–18). The pharmacokinetics of antimalarial drugs are affected by physiological changes of pregnancy,^13^ and lower blood concentration of lumefantrine on day 7 were reported in this patient group.^37^ Pregnant women with higher parasitaemia who are treated with AL need to be followed up closely. Dose optimisation, including courses longer than the recommended 3 days, might be necessary.

Although ASAQ, ASMQ, and DP showed higher efficacy than AL, the adjusted risk of treatment failure of ASSP was higher than AL (aHR 2·05, 95% CI 0·38–11·03, p=0·40), although not significant. ASSP efficacy was essentially equivalent to artesunate monotherapy (but given only for 3 days) in areas of sulfadoxine-pyrimethamine (SP) resistance. Considering the spread of SP resistance,^38^ ASSP provides a suboptimal therapeutic option for pregnant women compared with other ACTs. As was shown in non-pregnant populations,^39^ adding clindamycin to quinine resulted in efficacy equivalent to ABT, although poor tolerability of quinine could affect the adherence and acceptability to patients. Furthermore, clindamycin is not widely available in malaria-endemic countries.

Higher baseline asexual parasite density was associated with a higher risk of PCR-corrected treatment failure, as described in non-pregnant populations.^40^ This analysis found that nulliparous women were more likely to result in treatment failure in areas where transmission levels are moderate and high. This effect was most likely due to pregnancy-specific (ie, parity-dependent) immunity and was not observed in low transmission areas. These findings suggest that in an era of declining malaria endemicity, pregnant women might lose pregnancy-specific immunity and the risk of treatment failure might increase.^41^

The risk of recurrent falciparum malaria was high after AL and quinine monotherapy, as was expected from the shorter half-life and post-treatment prophylactic effect. Considering the cumulative effect of malaria recurrences on the fetus,^42^, ^43^ PCR-uncorrected efficacy is an important consideration when choosing the most appropriate drug for pregnant women, particularly in high transmission settings.

The use of QBT leads to a higher risk of gametocyte development than ABT because artemisinin derivatives have a gametocytocidal effect but quinine does not.^44^, ^45^ Lower bodyweight and higher baseline parasite density were associated with a higher risk of gametocytaemia on day 7, similar to non-pregnant populations.^46^, ^47^ Nulliparous women were at a higher risk of developing gametocytaemia, possibly due to lower pregnancy-specific immunity.

Quinine was associated with higher risks of abdominal pain, anorexia, dizziness, nausea, tinnitus, and vomiting than AL, limiting its practical use in real-life settings. Tolerability is important because pregnant women infected with malaria are generally less symptomatic than non-pregnant patients and are therefore less likely to accept adverse drug events. Poor adherence and patient discomfort could even be further exacerbated by morning sickness in the first trimester when QBT is recommended.

Among ACTs, adverse events were considered to be mainly due to the partner drug, and AL and DP were the better tolerated regimens. The risk of anorexia, nausea, vomiting, and dizziness was higher in ASAQ and ASMQ than in AL or DP, although none of the studies included in this pooled analysis were double-blinded. Pregnancy outcomes are described in detail by Saito and colleagues,^16^ but there was no difference among the ACTs tested (ie, AL, ASAQ, ASMQ, and DP).

This study has some limitations. Firstly, although this meta-analysis includes over 90% of patients that are available in the literature, some non-standard treatments were documented in only a small number of women. Nine studies^48^, ^49^, ^50^, ^51^, ^52^, ^53^, ^54^, ^55^, ^56^ (8% of individual patient data) were not included, but the pooled efficacy at the fixed timepoints was similar when aggregated data of those unshared studies were included (appendix p 8). Study designs and handling of indeterminate PCR or *P vivax* intercalated infection also did not affect our conclusions (appendix p 13–16), thereby confirming the robustness of our analysis as the most accurate global summary. Secondly, although the efficacy of ACTs shown in this study was satisfactory, careful consideration is needed when applying these results to specific settings because of variable patterns of drug resistance. Sensitivity to the partner drugs and immunity level differs depending on the location and study year, although a sensitivity analysis in which one study site was removed at a time did not change the conclusion of the meta-analysis (data not shown). The data from southeast Asia included in this study were collected before widespread artemisinin resistance in the area, but the spread of resistance in this region will affect the general recommendations for antimalarials, requiring alternative strategies and the safety of those formulations will have to be assessed in pregnant women. Thirdly, as study participants were mostly enrolled at antenatal clinics by screening for peripheral parasitaemia, the women included in this pooled analysis were more likely to be afebrile and with lower baseline parasite densities than patients in most antimalarial efficacy studies in non-pregnant populations.^46^ Thus, the efficacy at fixed time points might not be directly comparable to the results in non-pregnant populations or in settings where antenatal screening is not provided. Indeed, as baseline parasite density is a known risk factor for PCR-corrected treatment failure, the pooled efficacy at fixed timepoints in this study could have been overestimated. The lower efficacy of quinine and AL (under the dosing currently recommended by WHO)^14^ shown in this analysis could thus actually be worse for infections with higher parasitaemia. Nonetheless, the results of this study will be practically useful and more relevant than only including symptomatic patients because even asymptomatic parasitaemia leads to adverse pregnancy outcomes and most pregnant women with parasitaemia were asymptomatic.^1^, ^2^ Finally, evidence on the safety and efficacy of antimalarial treatment in the first trimester both in this meta-analysis and in the literature is scarce. Although safety of ACTs is reassuring^8^, ^9^ and there was no apparent effect of gestational age on the risk of PCR-corrected treatment efficacy indicated in this meta-analysis, collecting further evidence on the safety and efficacy of antimalarial treatment in first trimester women should be continued.

In conclusion, this meta-analysis, together with the evidence of safety shown in previous research,^8^, ^9^ provides compelling evidence that quinine monotherapy provides lower efficacy and tolerability than ACTs. Although the efficacy of QC was as high as that of ACTs, its practical use is discouraged considering the slow parasite clearance, the longer treatment period needed, the higher risk of gametocyte development, and poor tolerability. Although patients treated with AL had fewer acute adverse events, its efficacy was lower than other ACTs with the currently recommended dosing.^14^ In addition, the post-treatment prophylactic period of AL is shorter than other ACTs, leading to a higher risk of recurrences, which can cumulatively affect the fetus, particularly in higher transmission areas. ASAQ, ASMQ, and DP showed better efficacy compared with AL, but the tolerability of ASAQ and ASMQ was lower than AL. Considering the adverse effect of malaria on pregnant women and their offspring, all pregnant women infected with malaria, regardless of trimester, should be treated with the most effective drugs available.

## Data sharing

De-identified data are available from The WorldWide Antimalarial Resistance Network data repository.
