# Supplementary material for: Efficacy and tolerability of artemisinin-based and quinine-based treatments for uncomplicated falciparum malaria in pregnancy: a systematic review and individual patient data meta-analysis
Source: Lancet Infect Dis. 2020 Aug;20(8):943–52. doi: 10.1016/S1473-3099(20)30064-5 (PMC7391007; doi:10.1016/S1473-3099(20)30064-5)
Supplement: Supplementary appendix [file mmc1.pdf]

THE LANCET
Infectious Diseases

Supplementary appendix

This appendix formed part of the original submission and has been peer reviewed. We post it as supplied by the authors.

Supplement to: Saito M, Mansoor R, Kennon K, et al. Efficacy and tolerability of artemisinin-based and quinine-based treatments for uncomplicated falciparum malaria in pregnancy: a systematic review and individual patient data meta-analysis. *Lancet Infect Dis* 2020; published online April 29. https://doi.org/10.1016/S1473-3099(20)30064-5.

Web Appendix

Appendix Table 1. Design and quality of studies assessing PCR-confirmed treatment efficacy in pregnancy

Study [reference]	Country	Year	Study design	Randomisation	Allocation concealment	Blinding	Loss to follow-up for efficacy assessment	NOS	Reason for exclusion
**Shared studies**									
McGready, 2000[1]	Thailand	1995-1997	RCT	Block randomisation	Unclear	Open-label	0/66 (ASMQ)<br>0/42 (Q)	-	
McGready, 2001a[2]	Thailand	1997-2000	RCT	Block randomisation	Unclear	Open-label	19/65 (QC)<br>17/64 (AS)	-	
McGready, 2005[3]	Thailand	2002-2003	RCT	Computer-generated block randomisation	Sealed envelope	Open-label	0/39 (AAP)<br>0/42 (Q)	-	
Kalilani, 2007[4]	Malawi	2003-2004	RCT	Block randomisation	Sealed envelope	Open-label<br>Lab technicians were blinded.	8/47 (ASSP)<br>7/47 (SP)<br>5/47 (SP + azithromycin)	-	
McGready, 2008[5]	Thailand	2004-2006	RCT	Block randomisation	Sealed envelope	Open-label<br>Lab technicians and patient examiners were blinded.	5/125 (AL)<br>3/125 (AS)	-	
Piola, 2010[6]	Uganda	2006-2009	RCT	Computer-generated permuted block of eight	Sealed envelope	Open-label<br>Microscopists and clinical investigators were blinded.	10/152 (Q)<br>6/152 (AL)	-	
D'Alessandro, 2016[7,8]	Burkina Faso, Ghana, Malawi and Zambia	2010-2013	RCT	Block randomisation	Sealed envelope	Open-label<br>Lab technicians (interpretation of PCR) were blinded.	50/880 (AL)<br>51/842 (ASAQ)<br>94/853 (DP)<br>77/848 (ASMQ)	-	
Anvikar, 2018[9]	India	2010-2013	RCT	Permuted block of 12	Sealed envelope	Open-label	4/123 (ASMQ)<br>2/125 (ASSP)	-	
McGready, 2003a[10]	Thailand	2000-2001	PK study	Not applicable	Not applicable	Single-arm	8/24	8/8	
McGready, 2012[11]	Thailand	2008	PK study	No: assigned alternatively	Not applicable	Single-arm	0/20	8/8	
Rijken, 2011[12]	Thailand	2008-2009	PK study	Not applicable	Not applicable	Single-arm	2/24 (delivery)	8/8	

Appendix Table 1 continued.

Study [reference]	Country	Year	Study design	Randomisation	Allocation concealment	Blinding	Loss to follow-up for efficacy assessment	NOS	Reason for exclusion
Valea, 2014[13]	Burkina Faso	2008-2009	PK study	Not applicable	Not applicable	Single-arm	1/24	8/8	
Juma, 2014[14]	Kenia	2013-2014	PK study	Not applicable	Not applicable	Single-arm	INA	8/8	
Mosha, 2014[15]	Tanzania	2013	PK study	Not applicable	Not applicable	Single-arm	0/33	8/8	
Nyunt, 2016[16]	Uganda	2013-2014	PK study	Not applicable	Not applicable	Single-arm	0/30	8/8	
Ndiaye, 2011[17]	Senegal	2009	Single-arm	Not applicable	Not applicable	Single-arm	0/27	8/8	
McGready, 2003b[18]	Thailand	1999-2002	Observational cohort	Not applicable	Not applicable	Unclear	6/27	7/8	
Rijken, 2008[19]	Thailand	2006-2007	Observational cohort	Not applicable	Not applicable	Unclear	5/50 (delivery)	8/8	
Kalilani, 2013[20,21]	Malawi	2009-2011	Observational cohort	Not applicable	Not applicable	Unclear	120/450 (delivery) including those without malaria	7/8	

Appendix table 1 continued.

Study [reference]	Country	Year	Study design	Randomisation	Allocation concealment	Blinding	Loss to follow-up for efficacy assessment	NOS	Reason for exclusion
**Studies not published**									
NCT01054248[22]	Thailand	2010-2016	RCT	Computer generated permuted block of 15	sealed envelope	Open-label Microscopists were blinded.	INA	-	Not published
Onyamboko, 2015[23]	DRC	2013-2014	RCT	INA	INA	Open-label	INA	-	Not published
**IPD not sought**									
NCT02909712[24]	Tanzania	2016-	RCT	INA	INA	Assessors	INA	-	Study not completed
**Studies not included**									
Adam, 2004a[25]	Sudan	2002-2003	RCT	Yes, but the method is not specified.	Unclear	Unclear	7/25 (20mg/kg/day)<br>2/26 (30mg/kg/day)	-	Data not accessible
Adam, 2004c[26]	Sudan	1997-2001	Single-arm	Not applicable	Not applicable	Unclear	0/28	8/8	Data not accessible
Adam, 2006[27]	Sudan	2004-2005	Single-arm	Not applicable	Not applicable	Unclear	0/32	8/8	Data not accessible
Adam, 2012[28]	Sudan	2007-2008	PK study	Not applicable	Not applicable	Unclear	0/12	7/8	Data not accessible
Bounyasong, 2001[29]	Thailand	1995-1998	RCT	Yes, but the method is not specified.	Unclear	Unclear	1/30 (Q)<br>2/30 (ASSP)	-	No responses
Carmona-Fonseca, 2013[30]			RCT	Ballot system	Yes	Unclear	0/15 (AL)<br>0/15 (ASMQ)	-	Not shared by December 2018
Kaye, 2008[31]	Uganda	2006	RCT	Computer-generated random number	Sealed envelope	Open-label	5/57 (AL)<br>5/57 (CD)	-	Not shared by December 2018
Mutabingwa, 2009[32]	Tanzania	2004-2006	RCT	Block randomisation	Sealed envelope	Open-label Microscopists were blinded.	8/83 (ASAQ)<br>8/80 (AQSP)<br>4/81 (CD)<br>2/28 (SP)	-	Data not accessible
Mutagonda, 2017[33,34]	Tanzania	2014-2015	PK study	Not applicable	Not applicable	Unclear	10/92	8/8	Premature to be shared

AAP: artesunate-atovaquone-proguanil, AL: artemether-lumefantrine, AQ: amodiaquine, AS: artesunate, CHQ: chloroquine, CD: chlorproguanil-Dapsone, DP: dihydroartemisinin-piperaquine, DRC: Democratic Republic of the Congo, INA: information not available (registered trial). MQ: mefloquine, NOS: Newcastle-Ottawa scale. PK: pharmacokinetic, Q: quinine, QC: quinine-clindamycin, RCT: randomized controlled trial, SP: sulfadoxine-pyrimethamine.

Appendix Table 2. Information of the treatment used in the included studies

Study	Intermittent preventive treatment	Dosing of treatment
McGready, 2000[1]	Not applicable (not Sub-Saharan Africa)	Q (source unavailable): 10 mg salt/kg x 3 times/day for 7 days<br>AS (Guilin No 1 factory): 4mg/kg/day for 3 days<br>MQ (Lariam®), Roche): 25 mg base/kg given as 15 mg/kg on day 1 and 10 mg/kg on day 2
McGready, 2001a[2]	Not applicable (not Sub-Saharan Africa)	Q (Government Pharmaceutical Organization, Thailand)): 10 mg salt/kg x 3 times/day for 7 days<br>Clindamycin (Pharmacia & Upjohn)): 5 mg/kg x 3 times/day for 7 days<br>AS (Guilin No 1 factory): 2 mg/kg daily on days 0, 1, 2, 3 and 4 and 1 mg/kg daily on days 5 and 6 (total 10mg/kg)
McGready, 2005[3]	Not applicable (not Sub-Saharan Africa)	AS (Guilin Factory No 1): 4mg/kg/day for 3 days (50mg tablet)<br>AP (Malarone®, GSK): (atovaquone 20 mg/kg/day and 8 mg/kg/d) for 3 days (250+100mg tablet)<br>With 200mL of chocolate milk (8% fat)<br>Q (Government Pharmaceutical Organization, Thailand): 10 mg salt/kg x 3 times/day for 7 days
Kalilani, 2007[4]	The second dose of the same drug was administered at least 4 weeks after the first dose instead of IPTp	AS (source unavailable): 200mg/day for 3 days<br>SP (source unavailable): 1500+75mg as a single dose
McGready, 2008[5]	Not applicable (not Sub-Saharan Africa)	AL (Coartem®, Novartis): (80+480mg) x 2 times/day for 3 days<br>(at 0, 8, 24, 36, 48 and 60 h)<br>With 250mL of chocolate milk containing 7g of fat<br>AS (Guilin Pharmaceutical Factory,): 2mg/kg x 1 time/day for 7 days (50mg tablet)
Piola, 2010[6]	IPTp was interrupted until delivery	AL (Coartem®, Novartis): (80+480mg) x 2 times/day for 3 days (at 0, 8, 24, 36, 48 and 60 h)<br>With 200mL milk<br>Q (source unavailable): quinine hydrochloride 10 mg base/kg x 3 times/day for 7 days (300 mg of the base per tablet)
D'Alessandro, 2016[7,8]	IPTp was interrupted for 63 days of follow-up	AL (Coartem®, Novartis): (80+480mg) x 2times/day for 3 days<br>With recommendation to take high-fat food or drinks together<br>ASAQ (Winthrop®, Sanofi): (200+540mg) x 1 time/day for 3 days<br>ASMQ (Far-Manguinhos): (200+440mg/day) x 1 time/day for 3 days<br>DP (Eurartesim®, Sigma Tau): (120+960mg) x 1time/day for 3 days
Anvikar, 2018[9]	Not applicable (not Sub-Saharan Africa)	AS (source unavailable): 200mg/day for 3 days<br>MQ (source unavailable): 440mg/day for 3 days<br>AS (source unavailable): 200mg/day for 3 days<br>SP (source unavailable): 1500+75mg single dose on day1
McGready, 2003a[10]	Not applicable (not Sub-Saharan Africa)	AS (Guilin Factory No 1): 4mg/kg/day for 3 days (50mg tablet)<br>AP (Malarone®, Glaxo-Wellcome): (atovaquone 20 mg/kg/day and 8 mg/kg/d) for 3 days (250+100mg tablet)<br>With 200mL of chocolate milk (8% fat)
McGready, 2012[11]	Not applicable (not Sub-Saharan Africa)	Group 1: intravenous AS (Guilin Pharmaceutical Factory) 4mg/kg on day 0 followed by oral AS 4mg/kg for 6 days<br>Group 2: oral AS (Guilin Pharmaceutical Factory) 4mg/kg on day 0 followed by intravenous AS 4mg/kg on day 1 followed by oral artesunate 4mg/kg daily for 5 days

Appendix table 2 continued.

Study [reference]	Intermittent preventive treatment	Dosing of treatment
Rijken, 2011[12]	Not applicable (not Sub-Saharan Africa)	DP (Holley Pharm)<br>Total dosing of 6.5 mg/kg DHA and 51.2mg/kg PPQ over 3 days<br>The tablets (40+320mg) were divided to the nearest quarter
Valea, 2014[13]	IPTp-SP x 2	ASMQ (Farmanguinhos)<br>AS: 3.6mg/kg x 1 time/day for 3 days<br>MQ: 8mg/kg x 1 time/day for 3 days<br>(<50kg: 150+330mg/day<br>50-60kg: 200+440mg/day<br>>60kg: 250+550mg/day)
Juma, 2014[14]	Information not available	AL (source unavailable): (80+480mg) x 2 times/day for 3 days<br>(at hours 0, 8, 24, 36, 48 and 60)
Mosha, 2014[15]	Information not available	AL (Coartem®, Novartis): (80+480mg) x 2 times/day for 3 days<br>(at 0, 8, 24, 36, 48 and 60 h)<br>With 200mL of milk containing 4.5g of fat
Nyunt, 2016[16]	IPTp-SP x 2 (16-24w, 28-36 weeks)	AL (Coartem®, Novartis): (80+480mg) x 2 times/day for 3 days<br>With 200mL milk
Ndiaye, 2011[17]	Information not available	ASAQ (Coarsucam®, Sanofi)<br>AS: 200mg/day for 3 days<br>AQ: 540mg/day for 3 days
McGready, 2003b[18]	Not applicable (not Sub-Saharan Africa)	AS (Guilin Factory No 1): 4mg/kg/day for 3 days or 2mg/kg/day for 7 days (with a loading dose of 4 mg/kg in hyperparasitaemic patients with >4%)<br>AP (source unavailable): (atovaquone 20 mg/kg/day and 8 mg/kg/d) for 3 days
Rijken, 2008[19]	Not applicable (not Sub-Saharan Africa)	DP (Holley Pharm)<br>Total dosing of 6.5 mg/kg DHA and 51.2mg/kg PPQ over 3 days (40+320mg tablet)
Kalilani, 2013[20,21]	IPTp-SP x 3	AL (source unavailable): (80+480mg) x 2 times/day for 3 days<br>(at 0h, 8h, day2 AM, day2 PM, day3 AM, day3 PM)<br>With recommendation to take food (milk) together

AC: artesunate with clindamycin, AL: artemether-lumefantrine, AP: atovaquone-proguanil, AQ: amodiaquine, AS: artesunate, ASAQ: artesunate-amodiaquine, ASMQ: artesunate-mefloquine, DHA: dihydroartemisinin, DP: dihydroartemisinin-piperaquine, IPTp: intermittent preventive treatment in pregnancy, MQ: mefloquine, PPQ: piperaquine, Q: quinine, SP: sulfadoxine-pyrimethamine.

Appendix Table 3. Total mg/kg dose administrated (Median [Range]) for antimalarials

		Africa		Asia	
		N	Median [Range]	N	Median [Range]
AL	Artemether dose (mg/kg)	1142	8.9 [5.5- 14.1]	123	9.8 [7.4- 13.7]
	Lumefantrine dose (mg/kg)	1142	53.3 [32.7- 84.7]	123	58.8 [44.3- 82.3]
AAP	Artesunate dose (mg/kg)	No data		91	12.0 [11.5- 14.9]
	Atovaquone-proguanil dose (mg/kg)	No data		91	120.0 [87.0- 146.3]
AC	Artesunate dose (mg/kg)	No data		126	14.0 [13.1- 14.9]
	Clindamycin dose (mg/kg)	No data		126	126.0 [94.0- 165.8]
AS monotherapy	Artesunate dose (mg/kg)	No data		228	14.0 [8.0- 28.6]
ASAQ	Artesunate dose (mg/kg)	825	10.9 [5.6- 15.1]	No data	
	Amodiaquine dose (mg/kg)	825	29.5 [15.1- 40.8]	No data	
ASMQ	Artesunate dose (mg/kg)	832	11.1 [6.1- 17.8]	188	12.2 [10.0- 18.2]
	Mefloquine dose (mg/kg)	832	24.4 [13.5- 39.1]	188	26.1 [22.0- 40.0]
ASSP	Artesunate dose (mg/kg)	51	22.6 [18.9- 32.4]	122	13.3 [9.2- 19.4]
	SP dose (mg/kg)	51	28.3 [23.7-40.5]	122	33.3 [23.1-48.4]
DP	Dihydroartemisinin dose (mg/kg)	786	6.7 [3.1- 10.3]	82	6.5 [6.0- 7.3]
	Piperaquine dose (mg/kg)	786	53.3 [25.0- 82.3]	82	52.4 [48.0- 58.5]
Q monotherapy	Quinine dose (mg/kg)	131	222.8 [205.0- 245.3]	113	210.0 [143.2- 225.0]
QC	Quinine dose (mg/kg)	No data		67	210 [194.8- 225.0]
	Clindamycin dose (mg/kg)	No data		67	128.6 [98.4- 157.5]

N: Number of episodes.

AAP: artesunate with atovaquone-proguanil, AC: artesunate with clindamycin, AL: artemether-lumefantrine, AS: artesunate monotherapy, ASAQ: artesunate-amodiaquine, ASMQ: artesunate-mefloquine, ASSP: artesunate-sulfadoxine-pyrimethamine, CI: confidence interval, DP: dihydroartemisinin-piperaquine, PCR: polymerase chain reaction, Q: quinine monotherapy, QC: quinine with clindamycin.

Appendix Table 4. Heterogeneity of the PCR-corrected treatment efficacy for each treatment at fixed time points in each shared study

Treatment	Day 28		Day 42		Day 63	
	N	I^2	N	I^2	N	I^2
AL	10	68.6%	8	67.7%	6	80.2%
AAP	2	0.0%	2	24.8%	2	5.2%
AC	4	0.0%	2	70.5%	2	81.1%
AS	4	43.5%	4	46.0%	4	52.3%
ASAQ	7	0.0%	7	0.0%	7	44.3%
ASMQ	9	0.0%	9	72.7%	9	82.7%
ASSP	3	23.1%	1	-	1	-
DP	7	62.8%	7	76.5%	7	70.3%
Q	3	92.2%	3	92.4%	3	93.1%
QC	1	-	1	-	1	-

N: Number of study sites. -: only one study included.
AAP: artesunate with atovaquone-proguanil, AC: artesunate with clindamycin, AL: artemether-lumefantrine, AS: artesunate monotherapy, ASAQ: artesunate-amodiaquine, ASMQ: artesunate-mefloquine, ASSP: artesunate-sulfadoxine-pyrimethamine, CI: confidence interval, DP: dihydroartemisinin-piperaquine, PCR: polymerase chain reaction, Q: quinine monotherapy, QC: quinine with clindamycin.

Appendix Table 5. PCR-corrected treatment efficacy for each treatment at fixed time points in each shared and unshared study pooled by random effects

Treatment	PCR-corrected treatment success*					
	Day 28		Day 42		Day 63	
	N	% (95% CI)	N	% (95% CI)	N	% (95% CI)
AL	1311	96.9 (94.6–98.3)	929	95.5 (92.6–97.5)	598	93.6 (89.1–96.7)
AAP	58	99.9 (98.4–100)	49	98.9 (91.5–100)	40	98.5 (91.4–99.9)
AC	106	99.5 (97.4–99.9)	73	98.6 (85.4–100)	39	97.5 (71.4–100)
AS	169	95.9 (88.4–99.1)	140	95.6 (87.3–99.1)	91	95.3 (85.7–99.2)
ASAQ	881	99.6 (98.9–99.9)	782	99.2 (98.5–99.7)	611	98.8 (97.2–99.6)
ASMQ	1025	99.9 (99.6–100)	948	99.5 (98.3–99.9)	717	99.2 (97.0–99.9)
ASSP	191	99.3 (97.1–99.9)	120	99.2 (95.7–99.9)	113	99.2 (95.7–99.9)
DP	827	99.5 (98.3–99.9)	811	99.5 (97.5–99.9)	717	98.6 (96.1–99.7)
Q	205	93.5 (70.3–99.8)	164	86.8 (56.6–99.3)	128	84.9 (51.6–99.3)
QC	43	99.9 (97.6–100)	37	99.9 (97.0–100)	28	99.9 (96.3–100)

* Estimated by pooling the Kaplan-Meier survival estimates or proportions of patients with treatment success in each study by random effects method, including aggregated data from publication for the studies that IPD were not shared.
AAP: artesunate with atovaquone-proguanil, AC: artesunate with clindamycin, AL: artemether-lumefantrine, AS: artesunate monotherapy, ASAQ: artesunate-amodiaquine, ASMQ: artesunate-mefloquine, ASSP: artesunate-sulfadoxine-pyrimethamine, CI: confidence interval, DP: dihydroartemisinin-piperaquine, PCR: polymerase chain reaction, Q: quinine monotherapy, QC: quinine with clindamycin.

Appendix Table 6. Number of women with gametocytaemia after different treatment on different days after treatment stratified by the presence of gametocytaemia on day 0

Treatment	day 1	day 2	day 3	day 7	day 14	day 21	day 28
**Women without gametocytaemia on day 0**							
AL	13/1010	11/1024	7/951	3/945	1/998	0/945	0/1002
AAP	5/88	3/75	2/18	0/51	0/57	0/44	0/49
AC	1/103	1/95	4/66	3/77	1/86	1/86	1/67
AS	11/186	18/159	13/91	6/98	3/145	1/131	0/126
ASAQ	11/779	10/771	10/755	8/619	1/760	2/751	0/749
ASMQ	12/990	6/958	6/929	1/733	0/943	0/903	0/915
ASSP	1/115	1/117	1/114	0/98	0/112	0/115	0/114
DP	7/824	10/810	4/783	5/640	2/784	1/766	0/762
Q	6/199	5/184	11/163	11/166	4/169	1/159	0/152
QC	2/55	3/50	4/39	1/18	2/30	2/32	3/32
**Women with gametocytaemia on day 0**							
AL	9/36	10/40	14/38	7/33	4/39	1/36	0/45
AAP	3/4	3/4	1/2	0/1	1/1	0/2	No data
AC	7/11	7/11	8/9	8/12	3/9	2/9	1/13
AS	13/21	13/19	10/11	6/10	3/15	1/18	1/15
ASAQ	12/23	12/23	9/24	5/19	1/24	0/24	1/24
ASMQ	6/17	5/14	3/11	3/12	1/16	0/15	0/15
ASSP	3/4	1/4	1/4	1/3	1/4	1/4	0/4
DP	13/32	13/31	5/26	9/24	2/29	0/29	0/28
Q	7/17	9/15	6/15	6/15	2/12	1/10	1/11
QC	1/5	1/4	1/3	2/5	0/3	0/3	0/4

AAP: artesunate with atovaquone-proguanil, AL: artemether-lumefantrine, AS: artesunate monotherapy, ASAQ: artesunate-amodiaquine, ASMQ: artesunate-mefloquine, ASSP: artesunate-sulfadoxine-pyrimethamine, CI: confidence interval, DP: dihydroartemisinin-piperaquine, Q: quinine monotherapy, QC: quinine with clindamycin.

Appendix Table 7. Univariable and multivariable logistic regression of the risk of positive gametocytaemia on day 7 among women without gametocytaemia on day 0

Characteristic	Number positive/ number assessed	Univariable		Multivariable*	
		OR (95% CI)	p-value	OR (95% CI)	p-value
Treatment					
Artemisinin-based treatment	26/3261	Reference		Reference	
Quinine-based treatment	12/184	8.50 (2.55–28.33)	<0.001	7.38 (2.29–23.82)	0.001
EGA	38/3442	0.95 (0.90–1.01)	0.09		
Age group <20	17/1121	Reference			
20–24	9/1108	0.45 (0.20–1.06)	0.07		
25–29	4/655	0.32 (0.10–1.00)	0.05		
≥30	8/561	0.42 (0.17–1.05)	0.06		
Parity 0	21/1479	Reference		Reference	
1	6/722	0.49 (0.19–1.25)	0.14	0.41 (0.15–1.10)	0.08
≥2	11/1239	0.40 (0.19–0.87)	0.02	0.38 (0.17–0.89)	0.03
Weight (kg)	37/3426	0.92 (0.87–0.98)	0.01	0.93 (0.87–0.99)	0.02
Fever (temperature >37.5°C)					
Yes	5/297	1.11 (0.41–3.05)	0.83		
No	33/3130	Reference			
Haemoglobin on day 0 (g/dL)	38/3410	0.79 (0.63–0.99)	0.04		
Parasitaemia (log_{10}/µL)	38/3445	2.14 (1.46–3.16)	<0.001	1.82 (1.23–2.71)	0.003
Hyperparasitaemia					
Yes	1/36	1.42 (0.17–11.76)	0.74		
No	37/3409	Reference			
Mixed infection					
Yes	0/26	Reference			
No	38/3419	Reference			
Malaria transmission intensity					
Low	27/744	20.25 (3.56–115.11)	0.001	8.12 (1.39–47.55)	0.02
Moderate	7/1868	Reference		Reference	
High	4/833	2.06 (0.21–20.09)	0.53	3.15 (0.32–31.26)	0.33

*Adjusted for the variables in the column. Intraclass correlation 0.25. EGA: estimated gestational age, CI: confidence interval, OR: odds ratio.

Appendix Table 8. PCR–uncorrected treatment efficacy by treatment and intensity of malaria transmission at fixed time points in each shared study pooled by random effects

Treatment	PCR–uncorrected treatment success*					
	Day 28		Day 42		Day 63	
	N	% (95% CI)	N	% (95% CI)	N	% (95% CI)
**Low transmission**						
AL	174	92.7 (63.2–99.9)	157	87.7 (60.4–99.1)	110	78.9 (58.3–93.7)
AAP	59	99.9 (98.4–100.0)	50	98.9 (91.5–100.0)	41	93.4 (82.5–98.6)
AC	108	97.5 (83.7–99.9)	83	88.7 (65.6–98.8)	44	70.9 (40.3–94.8)
AS	177	90.9 (85.9–94.7)	147	89.9 (79.3–96.4)	95	83.3 (74.8–90.2)
ASAQ	25	99.9 (96.1–100.0)	25	99.9 (96.1–100.0)	9	99.9 (91.8–100.0)
ASMQ	177	99.2 (90.9–100.0)	171	99.2 (90.9–100.0)	160	98.2 (94.4–99.6)
ASSP	121	99.2 (95.7–99.9)	120	99.2 (95.7–99.9)	113	99.2 (95.7–99.9)
DP	71	92.1 (84.1–97.0)	63	82.5 (72.7–90.4)	44	76.1 (51.6–94.1)
Q	128	66.4 (42.2–88.6)	112	65.5 (41.2–88.2)	86	59.7 (36.1–84.2)
QC	43	99.9 (97.3–100.0)	37	97.5 (87.6–99.9)	28	94.2 (82.2–99.1)
**Moderate transmission**						
AL	726	92.2 (86.9–95.9)	559	84.7 (74.0–92.7)	402	77.4 (58.6–91.9)
ASAQ	470	99.2 (97.2–99.9)	458	97.6 (95.8–98.8)	425	93.0 (90.3–95.1)
ASMQ	491	99.4 (98.4–99.8)	475	99.1 (96.1–99.9)	389	87.2 (77.4–94.1)
ASSP	13	80.2 (55.8–95.9)	0		0	
DP	732	99.6 (98.7–99.9)	723	99.1 (97.4–99.8)	647	90.1 (84.1–94.5)
Q	111	73.0 (45.2–94.2)	76	62.1 (27.6–94.6)	57	54.4 (18.7–95.0)
**High transmission**						
AL	308	86.2 (82.1–89.8)	236	69.7 (57.5–81.1)	94	42.0 (36.2–48.4)
ASAQ	282	99.0 (97.0–99.7)	309	89.3 (82.1–94.5)	181	70.7 (57.5–82.8)
ASMQ	334	98.9 (94.5–99.9)	318	90.8 (83.6–95.7)	176	68.2 (44.5–89.2)
ASSP	25	96.0 (81.1–99.8)	0		0	
DP	56	99.9 (97.7–100.0)	55	99.9 (97.7–100.0)	46	86.3 (75.2–94.1)
Q	64	42.0 (27.7–59.8)	12	31.1 (18.3–49.6)	2[a]	27.8 (10.9–60.0)[a]

* Estimated by pooling the Kaplan-Meier survival estimates in each study by random effects method. [a]: including results between day 45 day 63.

AAP: artesunate with atovaquone-proguanil, AC: artesunate with clindamycin, AL: artemether-lumefantrine, AS: artesunate monotherapy, ASAQ: artesunate-amodiaquine, ASMQ: artesunate-mefloquine, ASSP: artesunate-sulfadoxine-pyrimethamine, CI: confidence interval, DP: dihydroartemisinin-piperaquine, PCR: polymerase chain reaction, Q: quinine monotherapy, QC: quinine with clindamycin.

Appendix Table 9. Heterogeneity of the PCR-uncorrected treatment efficacy by treatment and intensity of malaria transmission at fixed time points in each shared study

Treatment	Day 28		Day 42		Day 63	
	N	I^2	N	I^2	N	I^2
**Low transmission**						
AL	2	92.5%	2	93.3%	2	86.7%
AAP	2	0.0%	2	27.2%	2	0.0%
AC	4	67.1%	4	69.5%	4	72.4%
AS	5	0.0%	5	40.3%	5	13.7%
ASAQ	1	-	1	-	1	-
ASMQ	2	78.5%	2	78.5%	2	13.1%
ASSP	1	-	1	-	1	-
DP	2	0.0%	2	0.0%	2	75.9%
Q	4	87.6%	4	86.7%	4	86.0%
QC	1	-	1	-	1	-
**Moderate transmission**						
AL	6	69.4%	5	87.2%	4	94.9%
ASAQ	4	51.9%	4	1.0%	4	0.0%
ASMQ	5	9.4%	5	69.3%	5	79.6%
ASSP	1	-	0		0	
DP	5	32.9%	5	63.1%	5	74.7%
Q	5	86.9%	4	81.2%	3	74.7%
**High transmission**						
AL	3	0.0%	3	75.3%	2	0.0%
ASAQ	3	21.1%	3	62.3%	3	80.7%
ASMQ	3	69.5%	3	63.0%	3	93.6%
ASSP	1	-	0		0	
DP	1	-	1	-	1	-
Q	2	76.0%	2	47.6%	2	64.3%

N: Number of study sites. -: only one study included.
AAP: artesunate with atovaquone-proguanil, AC: artesunate with clindamycin, AL: artemether-lumefantrine, AS: artesunate monotherapy, ASAQ: artesunate-amodiaquine, ASMQ: artesunate-mefloquine, ASSP: artesunate-sulfadoxine-pyrimethamine, CI: confidence interval, DP: dihydroartemisinin-piperaquine, PCR: polymerase chain reaction, Q: quinine monotherapy, QC: quinine with clindamycin.

Appendix 1. Sensitivity analyses of risk factors for PCR-corrected treatment failure

Treatment effect was assessed by the piece-wise Cox model, which split the observation period into 0–28 days and >28 days, in order to satisfy the proportional hazards assumption.

In the multivariable analyses adjusted for parity and baseline parasitaemia (Appendix Table 1-1), quinine monotherapy remained associated with a higher risk of PCR-corrected treatment failure in both in day 0–28 (adjusted hazard ratio [aHR] 7·69, 95% confidence interval [CI] 3·11 to 18·99, p<0·001) and after day 28 (aHR 3·57, 95% CI 1·15 to 11·08, p=0·03) compared with AL. The risk of PCR-corrected treatment failure was lower in ASAQ (aHR 0·14, 95% CI 0·04 to 0·45, p=0·001), ASMQ (aHR 0·04, 95% CI 0·01 to 0·32, p=0·002) and DP (aHR 0·21, 95% CI 0·07 to 0·62, p=0·005) in day 0–28. The risk of failure after day 28 was also lower in ASAQ (aHR 0·44, 95% CI 0·20 to 0·97, p=0·04). The risk of treatment failure was lower in AC in day 0–28 (aHR 0·10, 95% CI 0·01 to 0·75, p=0·03) and higher in ASSP after day 28 (aHR 8·46, 95% CI 0·74 to 97·20, p=0·09), but these were based on relatively few observations.

Another pre-planned sensitivity analysis was conducted with *P. vivax* intercalated infection (n=233) being censored on the day of *P. vivax* appearance (following the current WHO guidelines for non-pregnant populations). The results were similar to the results of the other models (data not shown).

A post-hoc sensitivity analysis was conducted regarding the indeterminate PCR results (Appendix Table 1-2). One model regarded indeterminate PCR results as recrudescence (the worst-case scenario) and the other model regarded indeterminate PCR results as reinfection (the best case-scenario). The results were similar to our main analysis model.

Finally, to assess the difference of study designs, only randomised control trials (4571 episodes in eight studies) were included. The results were similar to the results of the other models (Appendix Table 1-3). Excluding one study site at a time also did not change the results (data not shown).

Appendix Table 1-1. Adjusted risk of PCR-corrected treatment failure for each treatment until and after day 28 by piece-wise Cox model

Treatment	Number of failure / includedł	Adjusted HR (95% CI)*	p-value
**Day 0–28**			
AL	40/1278	Reference	
AAP	0/91	No failure	
AC	1/142	0.10 (0.01–0.75)	0.03
AS	12/230	0.94 (0.45–1.97)	0.88
ASAQ	3/841	0.14 (0.04–0.45)	0.001
ASMQ	1/1028	0.04 (0.01–0.32)	0.002
ASSP	2/173	1.24 (0.18–8.84)	0.83
DP	4/874	0.21 (0.07–0.62)	0.005
Q	24/244	7.69 (3.11–18.99)	<0.001
QC	0/67	No failure	
**After day 28**			
AL	28/1036	Reference	
AAP	2/57	0.72 (0.14–3.83)	0.70
AC	5/100	0.80 (0.29–2.18)	0.66
AS	3/160	0.28 (0.08–0.96)	0.04
ASAQ	9/804	0.44 (0.20–0.97)	0.04
ASMQ	24/971	1.21 (0.67–2.19)	0.53
ASSP	2/135	8.46 (0.74–97.20)	0.09
DP	10/811	0.52 (0.24–1.16)	0.11
Q	7/174	3.57 (1.15–11.08)	0.03
QC	1/43	1.16 (0.10–13.09)	0.90

łNumber of women enrolled on day 0 or followed up on day 29. * Adjusted by parity, parasitaemia, previous antimalarial exposure and interaction between treatment and time. AAP: artesunate with atovaquone-proguanil, AC: artesunate with clindamycin, AL: artemether-lumefantrine, AS: artesunate monotherapy, ASAQ: artesunate-amodiaquine, ASMQ: artesunate-mefloquine, ASSP: artesunate-sulfadoxine-pyrimethamine, CI: confidence interval, DP: dihydroartemisinin-piperaquine, HR: hazard ratio, Q: quinine monotherapy, QC: quinine with clindamycin.

Appendix Table 1-2. Adjusted risk of PCR-corrected treatment failure assuming indeterminate PCR as recrudescence (worst-case scenario) or reinfection (best-case scenario) comparing with the main model

	Main model		Worst-case scenario		Best-case scenario	
Baseline characteristic	HR (95% CI)	p-value	HR (95% CI)	p-value	HR (95% CI)	p-value
Treatment						
AL	Reference		Reference		Reference	
AAP	0.31 (0.06–1.61)	0.17	0.35 (0.09–1.32)	0.12	0.32 (0.06–1.62)	0.17
AC	0.37 (0.15–0.91)	0.03	0.53 (0.24–1.15)	0.11	0.37 (0.15–0.90)	0.03
AS	0.64 (0.34–1.23)	0.18	0.81 (0.45–1.43)	0.47	0.62 (0.33–1.18)	0.15
ASAQ	0.27 (0.14–0.52)	<0.001	0.38 (0.24–0.59)	<0.001	0.28 (0.15–0.53)	<0.001
ASMQ	0.56 (0.34–0.94)	0.03	0.67 (0.45–0.98)	0.04	0.57 (0.34–0.94)	0.03
ASSP	2.05 (0.38–11.03)	0.40	2.56 (0.66–9.99)	0.18	1.94 (0.37–10.14)	0.43
DP	0.35 (0.18–0.68)	0.002	0.55 (0.34–0.87)	0.01	0.35 (0.18–0.68)	0.002
Q	6.11 (2.57–14.54)	<0.001	5.93 (3.09–11.36)	<0.001	5.74 (2.45–13.42)	<0.001
QC	0.48 (0.04–5.24)	0.55	0.34 (0.04–3.29)	0.35	0.49 (0.04–5.29)	0.55
Parity						
0	Reference		Reference		Reference	
1	0.59 (0.39–0.89)	0.01	0.71 (0.52–0.97)	0.03	0.59 (0.39–0.90)	0.01
≥2	0.62 (0.44–0.89)	0.009	0.62 (0.47–0.82)	<0.001	0.63 (0.44–0.89)	0.009
Parasitaemia (log10/µL)	1.93 (1.61–2.32)	<0.001	1.48 (1.29–1.71)	<0.001	1.93 (1.61–2.32)	<0.001

Adjusted by treatment, parity, parasitaemia and previous antimalarial exposure. AAP: artesunate with atovaquone-proguanil, AC: artesunate with clindamycin, AL: artemether-lumefantrine, AS: artesunate monotherapy, ASAQ: artesunate-amodiaquine, ASMQ: artesunate-mefloquine, ASSP: artesunate-sulfadoxine-pyrimethamine, CI: confidence interval, DP: dihydroartemisinin-piperaquine, HR: hazard ratio, Q: quinine monotherapy, QC: quinine with clindamycin.

Appendix Table 1-3. Adjusted risk of PCR-corrected treatment failure including only randomised control trials

	Main model		RCT only	
Baseline characteristic	HR (95% CI)	p-value	HR (95% CI)	p-value
Treatment				
AL	Reference		Reference	
AAP	0.31 (0.06–1.61)	0.17	0.42 (0.08–2.20)	0.30
AC	0.37 (0.15–0.91)	0.03	0.43 (0.17–1.07)	0.07
AS	0.64 (0.34–1.23)	0.18	0.64 (0.33–1.26)	0.20
ASAQ	0.27 (0.14–0.52)	<0.001	0.26 (0.14–0.50)	<0.001
ASMQ	0.56 (0.34–0.94)	0.03	0.55 (0.33–0.91)	0.02
ASSP	2.05 (0.38–11.03)	0.40	1.95 (0.39–9.83)	0.42
DP	0.35 (0.18–0.68)	0.002	0.24 (0.11–0.56)	<0.001
Q	6.11 (2.57–14.54)	<0.001	5.13 (2.06–12.78)	<0.001
QC	0.48 (0.04–5.24)	0.55	No failure	
Parity				
0	Reference		Reference	
1	0.59 (0.39–0.89)	0.01	0.57 (0.37–0.89)	0.01
≥2	0.62 (0.44–0.89)	0.009	0.55 (0.38–0.80)	0.002
Parasitaemia (log10/µL)	1.93 (1.61–2.32)	<0.001	1.89 (1.56–2.28)	<0.001

Adjusted by treatment, parity, parasitaemia and previous antimalarial exposure. AAP: artesunate with atovaquone-proguanil, AC: artesunate with clindamycin, AL: artemether-lumefantrine, AS: artesunate monotherapy, ASAQ: artesunate-amodiaquine, ASMQ: artesunate-mefloquine, ASSP: artesunate-sulfadoxine-pyrimethamine, CI: confidence interval, DP: dihydroartemisinin-piperaquine, HR: hazard ratio, Q: quinine monotherapy, QC: quinine with clindamycin RCT: randomised control trial.

Appendix 2. Dose response of AL

The dose-response was analysed only for AL, which was associated with a higher risk of treatment failure than other ACTs. The dose-response of quinine could not be investigated as quinine was administered based on body weight (i.e. patients received a very similar dose in mg/kg of body weight). Dose-response of AL (lumefantrine dose in mg per body weight in kg) was analysed by including women who completed the standard six-dose regimen over three days.

The standard dose (total 2880 mg of lumefantrine) was given to 1262 women regardless of body weight, following the study protocols of each study (11 study sites in eight studies). Fatty food was co-administered fully in four studies and partially in three studies but was not specified in one study. Twelve women did not have information on body weight and were thus excluded from the analyses. Overall, there was no apparent dose-response (aHR 0·99 per 1 mg/kg of lumefantrine, 95% CI 0·96 to 1·02, p=0·59) (Appendix Table 5). However, when the dose effect was assessed for each malaria transmission intensity area (p-value for interaction 0·09), a linear dose-response was found in the low transmission, but not in moderate or high transmission areas (Appendix Table 6). In low transmission areas, women receiving a higher dose (i.e. women with lower body weight) had a lower risk of PCR-corrected treatment failure (aHR 0·94 per 1 mg/kg of lumefantrine, 95% CI 0·88 to 1·00, p=0·048). Co-administration of fat was not associated with treatment outcome.

Appendix Table 2-1. Risk factors for PCR-corrected treatment failure among pregnant women who were treated with artemether-lumefantrine

Baseline characteristic	Number assessed (failure)	Univariable analysis		Multivariable analysis*	
		HR (95% CI)	p-value	HR (95% CI)	p-value
Dose for lumefantrine (mg/kg)	1,250 (67)	0.99 (0.96–1.02)	0.55	0.99 (0.96–1.02)	0.59
Age (years)	1,262 (68)	0.97 (0.93–1.02)	0.21		
Parity					
0	547 (33)	Reference			
1	250 (15)	0.81 (0.44–1.49)	0.49		
≥2	427 (18)	0.49 (0.27–0.88)	0.02		
Trimester					
1	2 (0)	No data			
2	861 (45)	Reference			
3	398 (23)	1.30 (0.77–2.17)	0.33		
Weight (kg)	1,250 (67)	1.01 (0.98–1.04)	0.62		
Parasitaemia (log10/µL)	1,262 (68)	2.00 (1.51–2.65)	<0.001	2.03 (1.54–2.69)	<0.001
Hyperparasitaemia					
Yes	24 (3)	2.79 (0.86–9.02)	0.09		
No	1,238 (65)	Reference			
Fever >37.5°C					
Yes	119 (12)	2.01 (1.04–3.85)	0.04		
No	1,097 (55)	Reference			
Haemoglobin (g/dL)	1,236 (67)	0.98 (0.82–1.17)	0.83		
Presence of gametocytes					
Yes	53 (5)	1.67 (0.66–4.22)	0.28		
No	1,173 (60)	Reference			
Mixed infection					
Yes	13 (1)	1.16 (0.16–8.55)	0.89		
No	1,249 (67)	Reference			
Intercalated vivax infection					
Yes	36 (4)	2.01 (0.64–6.28)	0.23		
No	1,226 (64)	Reference			
Fat intake					
Yes	344 (24)	1.25 (0.51–3.11)	0.63		
Partial (suggested)	871 (44)	Reference			
Transmission Intensity					
Low	200 (20)	1.64 (0.61–4.38)	0.32		
Moderate	760 (30)	0.82 (0.34–2.00)	0.66		
High	302 (18)	Reference			

*Adjusted for lumefantrine dose, baseline parasitaemia and previous treatment. HR: hazard ratio, CI: confidence interval.

Appendix Table 2-2. The dose–response of lumefantrine for the risk of PCR-corrected treatment failure by malaria transmission. Hazard ratios (HR) associated with 1 mg/kg change in lumefantrine dose are shown.

Malaria transmission	Number assessed (failure)	Multivariable analysis*	
		HR (95% CI)	p-value
Low	190 (19)	0.94 (0.88–1.00)	0.048
Moderate	758 (30)	1.00 (0.96–1.05)	0.84
High	302 (18)	1.04 (0.97–1.11)	0.29

*Adjusted for lumefantrine dose, baseline parasitaemia and previous treatment. HR: hazard ratio, CI: confidence interval. p-value for interaction: 0.088.

Appendix 3. Parasite clearance

Parasite positivity on day 1 and 2 was analysed by mixed-effects logistic regression, including those with recurrence without PCR. A total of 4830 women were assessed for parasitaemia on day 1 and included in the analysis. The unadjusted risk of positive parasitaemia on day 1 was higher in quinine monotherapy (odds ratio [OR] 4·20, 95% CI 2·69 to 6·56, p<0·001) and QC (OR 2·16, 95% CI 0·76 to 6·12, p=0·15) than AL (Appendix Table 2-1). The risk was lower in ASAQ (OR 0·38, 95% CI 0·28 to 0·53, p<0·001) and ASMQ (OR 0·51, 95% CI 0·39 to 0·65, p<0·001), DP (OR 0·43, 95% CI 0·31 to 0·59, p<0·001) and ASSP (OR 0·38, 95% CI 0·20 to 0·74, p=0·004). Other baseline characteristics associated with a higher risk of positive parasitaemia on day 1 included later gestational week (OR 1·03 per gestational week, 95% CI 1·01 to 1·04, p<0·001), younger age (OR 0·95 per year, 95% CI 0·94 to 0·97, p<0·001), primigravida/nullipara, presence of fever (OR 2·78, 95% CI 2·17 to 3·57, p<0·001), lower haemoglobin on day 0 (OR 0·90 per g/dL, 95% CI 0·85 to 0·95, p<0·001) and higher parasitaemia (OR 4·99 per 10-fold increase, 95% CI 4·37 to 5·69, p<0·001). When adjusted for estimated gestational age (EGA), parity, baseline haemoglobin and baseline parasitaemia, the risk of positive parasitaemia on day 1 was higher in quinine monotherapy (adjusted OR [aOR] 6·02, 95% CI 3·51 to 10·31, p<0·001), and lower in ASAQ (aOR 0·30, 95% CI 0·21 to 0·43, p<0·001), ASMQ (aOR 0·43, 95% CI 0·32 to 0·57, p<0·001), DP (aOR 0·35, 95% CI 0·25 to 0·50, p<0·001) and ASSP (aOR 0·29, 95% CI 0·13 to 0·61, p=0·001) than AL. After adjustment, later gestational week (aOR 1·02 per gestational week, 95% CI 1·00 to 1·03, p=0·04), lower haemoglobin on day 0 (aOR 0·92 per g/dL, 95% CI 0·86 to 0·99, p=0·02) and higher parasitaemia (aOR 5·02 per 10-fold increase, 95% CI 4·36 to 5·78, p<0·001) were associated with a higher risk of positive parasitaemia on day 1. The risk was lower in primiparous (aOR 0·69, 95% CI 0·54 to 0·90, p=0·006) or multiparous women (aOR 0·85, 95% CI 0·67 to 1·08, p=0·18) than nulliparous women.

A total of 4876 women were assessed for parasitaemia on day 2 and included in the analysis. The unadjusted risk of positive parasitaemia on day 2 was higher in quinine monotherapy (OR 12·06, 95% CI 5·93 to 24·52, p<0·001) and QC (OR 11·47, 95% CI 4·61 to 28·58, p<0·001) than AL (Appendix Table 2-2). The risks were not different in other ACTs. Other baseline characteristics associated with a higher risk of positive parasitaemia on day 2 included later gestational week (OR 1·02 per gestational week, 95% CI 1·00 to 1·04, p=0·02), higher parasitaemia (OR 3·07 per 10-fold increase, 95% CI 2·60 to 3·62, p<0·001), and presence of fever (OR 2·98, 95% CI 2·18 to 4·07, p<0·001). When adjusted for EGA, weight and baseline parasitaemia, the risk of positive parasitaemia on day 2 was higher in quinine monotherapy (aOR 33·64, 95% CI 13·63 to 83·00, p<0·001) and QC (aOR 22·92, 95% CI 7·90 to 66·50, p<0·001) than AL. The risk of positive parasitaemia on day 2 was

higher in DP but not significant (aOR 2·71, 95% CI 0·57 to 12·93, p=0·21). Higher body weight was associated with a lower risk of positive parasitaemia on day 2 in the adjusted analysis (aOR 0·97 per kg, 95% CI 0·94 to 0·99, p=0·01). After adjustment, later gestational week (aOR 1·04 per gestational week, 95% CI 1·01 to 1·06, p=0·005) and higher parasitaemia (aOR 3·60 per 10-fold increase, 95% CI 2·97 to 4·35, p<0·001) remained associated with a higher risk of positive parasitaemia on day 2.

On day 3, parasitaemia was negative in all patients treated with ASAQ (n=851), ASMQ (n=1021) and ASSP (n=174), while positive in AL (0·9%, 11/1214), DP (0·7%, 6/874), artesunate monotherapy (6·7%, 15/225), AC (9·0%, 11/122), AAP (2·2%, 2/90), quinine monotherapy (16·4%, 43/262) and QC (32·8%, 20/61). Further analyses were not conducted due to the small numbers.

Appendix Table 3-1. Risk factors for positive parasitaemia by microscopy on day 1

Baseline characteristic	Number of positive / assessed	Univariable		Multivariable*	
		OR (95% CI)	p-value	OR (95% CI)	p-value
Treatment					
AL	375/1,174	Reference		Reference	
AAP	72/92	0.86 (0.35–2.12)	0.74	0.95 (0.33–2.71)	0.92
AC	99/120	0.70 (0.35–1.40)	0.31	0.74 (0.33–1.66)	0.47
AS	181/227	0.99 (0.52–1.88)	0.98	0.95 (0.46–1.96)	0.88
ASAQ	70/837	0.38 (0.28–0.53)	<0.001	0.30 (0.21–0.43)	<0.001
ASMQ	164/1,014	0.51 (0.39–0.65)	<0.001	0.43 (0.32–0.57)	<0.001
ASSP	32/174	0.38 (0.20–0.74)	0.004	0.29 (0.13–0.61)	0.001
DP	120/866	0.43 (0.31–0.59)	<0.001	0.35 (0.25–0.50)	<0.001
Q	172/263	4.20 (2.69–6.56)	<0.001	6.02 (3.51–10.31)	<0.001
QC	53/63	2.16 (0.76–6.12)	0.15	1.92 (0.57–6.46)	0.29
Age (years)	1,338/4,830	0.95 (0.94–0.97)	<0.001		
Parity					
0	611/2,079	Reference		Reference	
1	251/1,018	0.54 (0.44–0.67)	<0.001	0.69 (0.54–0.90)	0.006
≥2	463/1,704	0.48 (0.40–0.59)	<0.001	0.85 (0.67–1.08)	0.18
EGA (weeks)	1,338/4,827	1.03 (1.01–1.04)	<0.001	1.02 (1.00–1.03)	0.04
Weight (kg)	1,326/4,815	1.00 (0.99–1.01)	0.49		
Parasitaemia (log10/µL)	1,338/4,830	4.99 (4.37–5.69)	<0.001	5.02 (4.36–5.78)	<0.001
Hyperparasitaemia					
Yes	59/70	12.02 (5.38–26.82)	<0.001		
No	1,279/4,760	Reference			
Fever					
Yes	279/476	2.78 (2.17–3.57)	<0.001		
No	1,036/4,279	Reference			
Haemoglobin (g/dL)	1,327/4,800	0.90 (0.85–0.95)	<0.001	0.92 (0.86–0.99)	0.02
Presence of gametocytes					
Yes	88/186	1.13 (0.76–1.67)	0.54		
No	1,219/4,557	Reference			
Mixed infection					
Yes	36/50	0.74 (0.36–1.51)	0.41		
No	1302/4,780	Reference			
Malaria transmission intensity					
Low	804/1,276	7.49 (1.43–39.41)	0.02		
Moderate	354/2,507	5.76 (1.10–30.22)	0.04		
High	180/1,047	Reference			

* Adjusted by treatment, parity, EGA, parasitaemia, and haemoglobin. Intraclass correlation: 0.51. AAP: artesunate with atovaquone-proguanil, AC: artesunate with clindamycin, AL: artemether-lumefantrine, AS: artesunate monotherapy, ASAQ: artesunate-amodiaquine, ASMQ: artesunate-mefloquine, ASSP: artesunate-sulfadoxine-pyrimethamine, CI: confidence interval, DP: dihydroartemisinin-piperaquine, EGA: estimated gestational age, OR: odds ratio, Q: quinine monotherapy, QC: quinine with clindamycin.

Appendix Table 3-2. Risk factors for positive parasitaemia by microscopy on day 2

Baseline characteristic	Number of positive / assessed	Univariable		Multivariable*	
		OR (95% CI)	p-value	OR (95% CI)	p-value
Treatment					
AL	45/1,212			Reference	
AAP	20/91	1.30 (0.54–3.15)	0.56	1.76 (0.60–5.13)	0.30
AC	47/120	1.60 (0.94–2.72)	0.08	1.79 (0.93–3.41)	0.08
AS	68/225	1.37 (0.83–2.25)	0.22	1.47 (0.83–2.60)	0.19
ASAQ	3/843	0.82 (0.18–3.69)	0.80	1.02 (0.19–5.61)	0.98
ASMQ	11/1,019	0.86 (0.33–2.27)	0.76	1.35 (0.44–4.11)	0.60
ASSP	4/175	0.77 (0.14–4.20)	0.77	1.23 (0.20–7.42)	0.82
DP	25/871	2.02 (0.48–8.53)	0.34	2.71 (0.57–12.93)	0.21
Q	93/259	12.06 (5.93–24.52)	<0.001	33.64 (13.63–83.00)	<0.001
QC	39/61	11.47 (4.61–28.58)	<0.001	22.92 (7.90–66.50)	<0.001
Age (years)	355/4876	0.99 (0.97–1.01)	0.21		
Parity					
0	124/2,108	Reference			
1	86/1,023	1.23 (0.87–1.73)	0.24		
≥2	144/1,714	0.85 (0.63–1.15)	0.30		
EGA (weeks)	355/4,873	1.02 (1.00–1.04)	0.02	1.04 (1.01–1.06)	0.005
Weight (kg)	349/4,860	0.99 (0.97–1.01)	0.22	0.97 (0.94–0.99)	0.01
Parasitaemia (log10/μL)	355/4,876	3.07 (2.60–3.62)	<0.001	3.60 (2.97–4.35)	<0.001
Hyperparasitaemia					
Yes	30/72	5.39 (2.82–10.33)	<0.001		
No	325/4,804	Reference			
Fever					
Yes	117/476	2.98 (2.18–4.07)	<0.001		
No	235/4,295	Reference			
Haemoglobin (g/dL)	352/4,843	1.07 (0.98–1.16)	0.11		
Presence of gametocytes					
Yes	31/190	0.96 (0.61–1.52)	0.87		
No	314/4,599	Reference			
Mixed infection					
Yes	16/48	0.99 (0.52–1.89)	0.98		
No	339/4,828	Reference			
Malaria transmission intensity					
Low	333/1,272	12.84 (0.81–204.21)	0.07		
Moderate	17/2521	12.50 (0.77–203.93)	0.08		
High	5/1,083	Reference			

* Adjusted by treatment, EGA, weight, and parasitaemia and previous antimalarial treatment. Intraclass correlation: 0.55. AAP: artesunate with atovaquone-proguanil, AC: artesunate with clindamycin, AL: artemether-lumefantrine, AS: artesunate monotherapy, ASAQ: artesunate-amodiaquine, ASMQ: artesunate-mefloquine, ASSP: artesunate-sulfadoxine-pyrimethamine, CI: confidence interval, DP: dihydroartemisinin-piperaquine, EGA: estimated gestational age, OR: odds ratio, Q: quinine monotherapy, QC: quinine with clindamycin.

Appendix 4. Fever clearance

Body temperature was assessed on day 0 (17 studies), 1 (14 studies), 2 (14 studies) and 3 (15 studies). Estimated using random effects including those with recurrent infection not genotyped (or indeterminant), the pooled proportion of febrile women (>37·5 °C) was only 15.1% (95% CI 10.5% to 20.3%, n=4969) at inclusion. After treatment, a pooled proportion of 1.8% (95% CI 0.9% to 3.0%, 103/4509) women were febrile on day 1, but the majority were afebrile on day 2 (0.0%, 95% CI 0.0% to 0.1%, 17/4420) and day 3 (0.0%, 95% CI 0.0% to 0.0%, 13/4207). The pooled proportions of febrile patients by treatment on day 0, 1, 2 and 3 are summarized in Appendix Table 4-1. Further analyses were not conducted because of the small number of febrile women and different practices on antipyretic drug use in different studies.

Appendix Table 4-1. Proportions of febrile women on day 0 to day 3 by different treatment pooled by random effects

Treatment	Day 0		Day 1		Day 2		Day 3	
	N	% (95% CI)	N	% (95% CI)	N	% (95% CI)	N	% (95% CI)
AL	1256	13.7 (7.0–22.1)	1159	2.1 (0.1–5.7)	1158	0.0 (0.0–0.2)	1129	0.0 (0.0–0.3)
AAP	92	24.7 (7.6–46.9)	81	10.9 (1.4–25.9)	77	0.0 (0.0–2.5)	24	0.0 (0.0–7.9)
AC	135	14.1 (0.5–37.0)	2	No data	3	No data	2	No data
AS	233	26.2 (13.7–40.7)	213	6.2 (3.0–10.1)	194	0.0 (0.0–0.6)	124	0.1 (0.0–2.7)
ASAQ	857	7.3 (2.3–14.6)	837	0.1 (0.0–1.3)	825	0.0 (0.0–0.4)	814	0.0 (0.0–0.2)
ASMQ	1049	8.2 (4.1–13.3)	1011	1.5 (0.2–3.7)	976	0.3 (0.0–0.9)	946	0.0 (0.0–0.5)
ASSP	121	25.6 (18.7–34.1)	119	4.2 (1.8–9.5)	121	2.5 (0.8–7.0)	118	1.7 (0.5–6.0)
DP	890	4.5 (1.2–9.5)	821	0.6 (0.0–2.2)	817	0.0 (0.0–0.3)	811	0.0 (0.0–0.0)
Q	264	17.8 (13.1–22.9)	204	2.9 (0.0–8.4)	196	0.1 (0.0–1.7)	203	0.0 (0.0–0.9)
QC	67	19.7 (11.9–30.8)	62	6.5 (2.5–15.4)	53	1.9 (0.3–9.9)	36	2.8 (0.5–14.2)

AAP: artesunate with atovaquone-proguanil, AL: artemether-lumefantrine, AS: artesunate monotherapy, ASAQ: artesunate-amodiaquine, ASMQ: artesunate-mefloquine, ASSP: artesunate-sulfadoxine-pyrimethamine, CI: confidence interval, DP: dihydroartemisinin-piperaquine, Q: quinine monotherapy, QC: quinine with clindamycin.

Appendix 5. Acute adverse events

There was six maternal deaths, one of which was due to severe malaria which developed after treatment with ASSP.[9] The deaths of the other five women were considered not to be associated with malaria.

The symptoms that developed within a week after starting treatment were analysed by mixed-effects logistic regression on nine commonly assessed symptoms. The pooled analysis below was conducted by including only studies in which each symptom was actively assessed (Appendix Table 5-1).

A total of 4300 women were actively assessed for the presence of abdominal pain. The risk of abdominal pain was higher in ASAQ (aOR 2·03, 95% CI 1·40 to 2·96, p<0·001), quinine (aOR 1·81, 95% CI 1·07 to 3·04, p=0·03), and ASMQ (aOR 1·44, 95% CI 1·03 to 2·02, p=0·03) than AL (Appendix Table 5-2). No other baseline characteristics (except the presence of headache on day 0) were associated with the risk of headache.

A total of 4376 women were actively assessed for the presence of anorexia. The risk of anorexia was higher in quinine (aOR 4·29, 95% CI 1·99 to 9·23, p<0·001), ASAQ (aOR 3·88, 95% CI 2·36 to 6·38, p<0·001), QC (aOR 3·41, 95% CI 1·19 to 9·79, p=0·02), and ASMQ (aOR 2·41, 95% CI 1·53 to 3·79, p<0·001) than AL (Appendix Table 5-3). Higher parasitaemia was associated with an increased risk of anorexia (aOR 1·23 per 10-fold increase, 95% CI 1·07 to 1·40, p=0·003). Younger age (< 20 years old) was associated with a higher risk of anorexia.

A total of 4622 women were actively assessed for the presence of dizziness. The risk of dizziness was higher in ASMQ (aOR 17·03, 95% CI 11·08 to 26·16, p<0·001), ASAQ (aOR 16·04, 95% CI 10·21 to 25·18, p<0·001), quinine (aOR 10·25, 95% CI 6·08 to 17·28, p<0·001), ASSP (aOR 8·82, 95% CI 3·40 to 22·85, p<0·001), QC (aOR 8·22, 95% CI 2·86 to 23·68, p<0·001), AAP (aOR 4·60, 95% CI 1·55 to 13·65, p=0·01), and DP (aOR 2·58, 95% CI 1·43 to 4·67, p=0·02) than AL (Appendix Table 5-4). Hyperparasitaemia (aOR 2·26, 95% CI 1·08 to 4·74, p=0·03) was associated with a higher risk of dizziness. EGA was associated with decreased risk of dizziness (aOR 0·98 per week, 95% CI 0·97 to 1·00, p=0·04). Compared with nulliparous women, multiparous women (aOR 1·33, 95% CI 1·09 to 1·63, p=0·01) were more likely to have dizziness but similar in primiparous women (aOR 1·14, 95% CI 0·90 to 1·45, p=0·27).

A total of 4246 women were actively assessed for the presence of fatigue. The risk of fatigue was particularly higher in ASAQ (aOR 12·65, 95% CI 8·70 to 18·38, p<0·001) than AL (Appendix Table 5-5). To a lesser extent, the risk was also higher in ASSP (aOR 3·93, 95% CI 1·97 to 7·84, p<0·001), ASMQ (aOR 2·62, 95% CI

1·84 to 3·71, p<0·001), quinine (aOR 2·43, 95% CI 1·02 to 5·78, p=0·05), and DP (aOR 2·16, 95% CI 1·48 to 3·16, p<0·001). Women of higher age (aOR 1·03 per year, 95% CI 1·01 to 1·04, p<0·001) and lower baseline haemoglobin (aOR 0·93 per g/dL, 95% CI 0·87 to 1·00, p=0·04) were more likely to develop fatigue.

A total of 4623 women were actively assessed for the presence of headache. The risk of headache was higher in ASAQ (aOR 1·56, 95% CI 1·14 to 2·14, p=0·01) than AL (Appendix Table 5-6). No other baseline characteristics (except the presence of headache on day 0) were associated with the risk of headache.

A total of 4299 women were actively assessed for the presence of musculoskeletal pain. The risk of musculoskeletal pain was uniquely higher in ASAQ (aOR 2·83, 95% CI 1·88 to 4·24, p<0·001) than AL (Appendix Table 5-7). Women with fever on day 0 (aOR 1·58, 95% CI 1·11 to 2·24, p=0·01) and women in later gestational week (aOR 1·02 per week, 95% CI 1·00 to 1·04, p=0·03) were more likely to have musculoskeletal pain. Compared with nulliparous women, multiparous women (aOR 1·45, 95% CI 1·14 to 1·85, p=0·003) were more likely to have musculoskeletal pain but similar in primiparous women (aOR 1·08, 95% CI 0·80 to 1·45, p=0·61).

A total of 4609 women were actively assessed for the presence of nausea. The risk of nausea was higher in quinine (aOR 7·29, 95% CI 3·78 to 14·08, p<0·001), ASAQ (aOR 6·04, 95% CI 3·68 to 9·91, p<0·001), ASMQ (aOR 5·68, 95% CI 3·58 to 9·00, p<0·001), AAP (4·70, 95% CI 1·83 to 12·07, p=0·001), and DP (aOR 2·88, 95% CI 1·70 to 4·88, p<0·001) than AL (Appendix Table 5-8). The risk of nausea was higher in QC (aOR 2·07, 95% CI 0·80 to 5·39, p=0·14) but not significant. Women of higher age (aOR 1·02 per year, 95% CI 1·01 to 1·04, p=0·01) and women in earlier pregnancy (aOR 0·98, 95% CI 0·97 to 1·00, p=0·05) were more likely to develop nausea.

A total of 4516 women were actively assessed for the presence of tinnitus. The risk of tinnitus was higher in quinine (aOR 249·84, 95% CI 80·90 to 771·56, p<0·001) and QC (aOR 71·91, 95% CI 19·45 to 265·86, p<0·001) than AL (Appendix Table 5-9). Among ACTs, the risk was higher in AAP (aOR 6·29, 95% CI 1·33 to 29·86, p=0·02), ASMQ (aOR 6·22, 95% CI 1·56 to 24·82, p=0·01), DP (aOR 5·75, 95% CI 1·19 to 27·77, p=0·03), and ASAQ (aOR 4·24, 95% CI 0·89 to 20·29, p=0·07) than AL. No other baseline characteristics (except the presence of tinnitus on day 0) were associated with the risk of tinnitus.

A total of 4618 women were actively assessed for the presence of vomiting. The risk of vomiting was higher in ASAQ (aOR 11·56, 95% CI 7·04 to 19·00), ASMQ (aOR 10·34, 95% CI 6·38 to 16·76, p<0·001), quinine (aOR 9·61, 95% CI 4·70 to 19·63, p<0·001) and QC (aOR 8·73, 95% CI 3·00 to 25·37, p<0·001) than AL

(Appendix Table 5-10). To a lesser extent, the risk was also higher in AAP (aOR 4·96, 95% CI 1·667 to 14·80, p=0·004), DP (aOR 4·24, 95% CI 2·49 to 7·21, p<0·001), and AS (aOR 2·07, 95% CI 1·03 to 4·17, p=0·04). Women in later gestational week (aOR 0·98 per week, 95% CI 0·96 to 1·00, p=0·03) were associated with a lower risk of vomiting.

Appendix Table 5-1. Studies included in the IPD meta-analysis for each symptom

Study	Abdominal pain	Anorexia	Dizziness	Headache	Musculoskeletal pain	Nausea	Tinnitus	Vomiting	Fatigue
McGready, 2000[1]	Yes	Yes	Yes	Yes	Yes	Yes	Yes	Yes	No
McGready, 2001a[2]	Yes	Yes	Yes	Yes	Yes	Yes	Yes	Yes	No
McGready, 2005[3]	Yes	Yes	Yes	Yes	Yes	Yes	Yes	Yes	No
Kalilani, 2007[4]	No	No	No	No	No	No	No	No	No
McGready, 2008[5]	Yes	Yes	Yes	Yes	Yes	Yes	Yes	Yes	Yes
Piola, 2010[6]	Yes	Yes	Yes	Yes	Yes	Yes	Yes	Yes	Yes
D'Alessandro, 2016[7,8]	Yes	Yes	Yes	Yes	Yes	Yes	Yes	Yes	Yes
Anvikar, 2018[9]	No	No	Yes	Yes	No	Yes	Yes	Yes	Yes
McGready, 2003a[10]	Yes	Yes	Yes	Yes	Yes	Yes	Yes	Yes	No
McGready, 2012[11]	Yes	Yes	Yes	Yes	Yes	Yes	Yes	Yes	Yes
Rijken, 2011[12]	Yes	Yes	Yes	Yes	Yes	Yes	Yes	Yes	Yes
Valea, 2014[13]	No	No	No	No	No	No	No	No	No
Juma, 2014[14]	No	Yes	Yes	Yes	No	Yes	No	Yes	Yes
Mosha, 2014[15]	No	No	No	No	No	No	No	No	No
Nyunt, 2016[16]	Yes	Yes	No	Yes	Yes	Yes	No	Yes	No
Ndiaye, 2011[17]	No	Yes	Yes	Yes	No	No	No	Yes	Yes
McGready, 2003b[18]	Yes	Yes	Yes	Yes	Yes	Yes	Yes	Yes	No
Rijken, 2008[19]	No	No	No	No	No	No	No	No	No
Kalilani, 2013[20,21]	No	No	No	No	No	No	No	No	No

Appendix Table 5-2. Univariable and multivariable logistic regression of the risk of developing abdominal pain on day 1–7

Baseline characteristic	Number of positive / assessed	Univariable		Multivariable*	
		OR (95% CI)	p-value	OR (95% CI)	p-value
Treatment					
AL	103/1177	Reference		Reference	
AAP	21/73	2.24 (0.83–6.04)	0.11	2.26 (0.80–6.34)	0.12
AC	0/9	Omitted			
AS	49/200	1.19 (0.64–2.22)	0.58	1.19 (0.64–2.23)	0.58
ASAQ	99/827	2.01 (1.39–2.92)	<0.001	2.03 (1.40–2.96)	<0.001
ASMQ	133/891	1.43 (1.02–2.00)	0.04	1.44 (1.03–2.02)	0.03
ASSP	5/5	Omitted			
DP	80/831	1.05 (0.72–1.54)	0.79	1.08 (0.74–1.58)	0.69
Q	54/228	1.78 (1.07–2.98)	0.03	1.81 (1.07–3.04)	0.03
QC	18/59	1.33 (0.49–3.59)	0.58	1.32 (0.48–3.62)	0.59
Baseline abdominal pain					
Yes	265/774	5.56 (4.46–6.94)	<0.001	5.73 (4.59–7.16)	<0.001
No	287/3516	Reference		Reference	
Age (years)	562/4300	1.00 (0.98–1.01)	0.82		
Parity					
0	233/1849	Reference			
1	120/895	0.93 (0.71–1.20)	0.57		
≥2	209/1549	0.90 (0.72–1.13)	0.36		
EGA (weeks)	562/4297	0.99 (0.97–1.01)	0.32		
Weight (kg)	562/4283	0.99 (0.97–1.00)	0.05		
Parasitaemia (log10/µL)	562/4300	1.15 (1.02–1.29)	0.02		
Hyperparasitaemia					
Yes	9/47	0.86 (0.38–1.98)	0.73		
No	553/4253	Reference			
Fever					
Yes	78/350	1.25 (0.90–1.72)	0.18		
No	479/3906	Reference			
Haemoglobin (g/dL)	559/4262	0.97 (0.91–1.04)	0.45		
Presence of gametocytes					
Yes	23/154	0.78 (0.47–1.29)	0.33		
No	536/4138	Reference			
Mixed infection					
Yes	7/32	0.92 (0.35–2.44)	0.87		
No	555/4268	Reference			
Malaria transmission intensity					
Low	198/768	1.43 (0.70–2.90)	0.32	1.43 (0.70–2.90)	0.32
Moderate	219/2476	Reference		Reference	
High	145/1056	3.41 (1.89–6.15)	<0.001	3.41 (1.89–6.15)	<0.001

* Adjusted by variables in the column. Intraclass correlation: 0.19. AAP: artesunate with atovaquone-proguanil, AC: artesunate with clindamycin, AL: artemether-lumefantrine, AS: artesunate monotherapy, ASAQ: artesunate-amodiaquine, ASMQ: artesunate-mefloquine, ASSP: artesunate-sulfadoxine-pyrimethamine, CI: confidence interval, DP: dihydroartemisinin-piperaquine, EGA: estimated gestational age, OR: odds ratio, Q: quinine monotherapy, QC: quinine with clindamycin.

Appendix Table 5-3. Univariable and multivariable logistic regression of the risk of developing anorexia on day 1–7

	Number of positive /	Univariable		Multivariable*	
Baseline characteristic	assessed	OR (95% CI)	p-value	OR (95% CI)	p-value
Treatment					
AL	116/1228	Reference		Reference	
AAP	48/79	3.30 (1.02–10.71)	0.05	2.96 (0.95–9.20)	0.06
AC	2/9	Omitted			
AS	133/206	1.29 (0.71–2.35)	0.40	1.34 (0.73–2.44)	0.34
ASAQ	114/853	3.80 (2.31–6.25)	<0.001	3.88 (2.36–6.38)	<0.001
ASMQ	117/881	2.39 (1.52–3.77)	<0.001	2.41 (1.53–3.79)	<0.001
ASSP	No data	Omitted			
DP	62/829	1.33 (0.79–2.23)	0.28	1.32 (0.79–2.22)	0.29
Q	57/227	4.66 (2.15–10.10)	<0.001	4.29 (1.99–9.23)	<0.001
QC	49/64	3.34 (1.17–9.49)	0.02	3.41 (1.19–9.79)	0.02
Baseline anorexia					
Yes	441/798	9.20 (7.20–11.75)	<0.001	8.80 (6.86–11.28)	<0.001
No	255/3576	Reference		Reference	
Age (years)					
<20	142/1470	Reference		Reference	
20–24	207/1332	1.50 (1.12–2.02)	0.01	1.61 (1.19–2.16)	0.002
25–29	153/838	1.43 (1.04–1.99)	0.03	1.55 (1.11–2.16)	0.01
≥30	196/736	1.65 (1.19–2.27)	0.002	1.78 (1.28–2.47)	0.001
Parity					
0	222/1867	Reference			
1	146/907	1.09 (0.81–1.47)	0.55		
≥2	310/1569	1.29 (1.01–1.66)	0.05		
EGA (weeks)	698/4373	1.01 (0.99–1.03)	0.38		
Weight (kg)	696/4359	0.99 (0.98–1.01)	0.26		
Parasitaemia (log10/µL)	698/4376	1.20 (1.05–1.36)	0.01	1.23 (1.07–1.40)	0.003
Hyperparasitaemia					
Yes	24/53	2.64 (1.20–5.83)	0.02		
No	674/4323	Reference			
Fever					
Yes	142/375	1.50 (1.07–2.11)	0.02		
No	552/3953	Reference			
Haemoglobin (g/dL)	695/4339	1.00 (0.92–1.07)	0.92		
Presence of gametocytes					
Yes	44/155	1.17 (0.71–1.95)	0.54		
No	631/4187	Reference			
Mixed infection					
Yes	17/35	0.91 (0.36–2.25)	0.83		
No	681/4341	Reference			
Malaria transmission intensity					
Low	405/804	3.01 (1.09–8.32)	0.03	2.83 (1.05–7.65)	0.04
Moderate	161/2517	Reference		Reference	
High	132/1055	3.49 (1.93–6.33)	<0.001	3.32 (1.82–6.04)	<0.001

* Adjusted by variables in the column. Intraclass correlation: 0.30. AAP: artesunate with atovaquone-proguanil, AC: artesunate with clindamycin, AL: artemether-lumefantrine, AS: artesunate monotherapy, ASAQ: artesunate-amodiaquine, ASMQ: artesunate-mefloquine, ASSP: artesunate-sulfadoxine-pyrimethamine, CI: confidence interval, DP: dihydroartemisinin-piperaquine, EGA: estimated gestational age, OR: odds ratio, Q: quinine monotherapy, QC: quinine with clindamycin.

Appendix Table 5-4. Univariable and multivariable logistic regression of the risk of developing dizziness on day 1–7

Baseline characteristic	Number of positive / assessed	Univariable		Multivariable*	
		OR (95% CI)	p-value	OR (95% CI)	p-value
Treatment					
AL	106/1198	Reference		Reference	
AAP	50/78	4.36 (1.41–13.51)	0.01	4.60 (1.55–13.65)	0.01
AC	3/11	0.81 (0.13–5.12)	0.82	0.90 (0.15–5.48)	0.91
AS	121/206	1.30 (0.76–2.24)	0.34	1.37 (0.79–2.36)	0.26
ASAQ	217/857	16.00 (10.21–25.08)	<0.001	16.04 (10.21–25.18)	<0.001
ASMQ	324/1025	17.08 (11.12–26.21)	<0.001	17.03 (11.08–26.16)	<0.001
ASSP	10/121	8.96 (3.47–23.17)	<0.001	8.82 (3.40–22.85)	<0.001
DP	36/834	2.60 (1.44–4.70)	0.002	2.58 (1.43–4.67)	0.002
Q	143/228	9.08 (5.46–15.09)	<0.001	10.25 (6.08–17.28)	<0.001
QC	55/64	7.89 (2.74–22.70)	<0.001	8.22 (2.86–23.68)	<0.001
Baseline dizziness					
Yes	351/494	5.01 (3.73–6.74)	<0.001	4.79 (3.54–6.47)	<0.001
No	708/4122	Reference		Reference	
Age (years)	1065/4622	1.02 (1.00–1.03)	0.04		
Parity					
0	358/1999	Reference		Reference	
1	223/974	1.15 (0.91–1.45)	0.26	1.14 (0.90–1.45)	0.27
≥2	470/1618	1.30 (1.07–1.59)	0.01	1.33 (1.09–1.63)	0.01
EGA (weeks)	1064/4619	0.99 (0.97–1.00)	0.14	0.98 (0.97–1.00)	0.04
Weight (kg)	1064/4605	0.99 (0.98–1.00)	0.16		
Parasitaemia (log10/µL)	1065/4622	1.02 (0.92–1.14)	0.65		
Hyperparasitaemia					
Yes	28/60	2.19 (1.06–4.54)	0.03	2.26 (1.08–4.74)	0.03
No	1037/4562	Reference		Reference	
Fever					
Yes	174/438	1.15 (0.88–1.51)	0.32		
No	889/4164	Reference			
Haemoglobin (g/dL)	1055/4581	1.04 (0.97–1.10)	0.27		
Presence of gametocytes					
Yes	57/161	0.82 (0.53–1.26)	0.37		
No	985/4423	Reference			
Mixed infection					
Yes	21/35	1.84 (0.78–4.35)	0.16		
No	1044/4587	Reference			
Malaria transmission intensity					
Low	522/1060	3.10 (1.79–5.36)	<0.001	3.14 (1.79–5.50)	<0.001
Moderate	210/2535	Reference		Reference	
High	333/1027	2.99 (1.47–6.06)	0.002	2.92 (1.44–5.95)	0.003

* Adjusted by variables in the column. Intraclass correlation: 0.35. AAP: artesunate with atovaquone-proguanil, AC: artesunate with clindamycin, AL: artemether-lumefantrine, AS: artesunate monotherapy, ASAQ: artesunate-amodiaquine, ASMQ: artesunate-mefloquine, ASSP: artesunate-sulfadoxine-pyrimethamine, CI: confidence interval, DP: dihydroartemisinin-piperaquine, EGA: estimated gestational age, OR: odds ratio, Q: quinine monotherapy, QC: quinine with clindamycin.

Appendix Table 5-5. Univariable and multivariable logistic regression of the risk of developing fatigue on day 1–7

Baseline characteristic	Number of positive / assessed	Univariable		Multivariable*	
		OR (95% CI)	p-value	OR (95% CI)	p-value
Treatment					
AL	139/1199	Reference		Reference	
AAP	No data	Omitted			
AC	0/3	Omitted			
AS	67/141	1.01 (0.58–1.78)	0.97	0.97 (0.55–1.70)	0.91
ASAQ	309/853	12.74 (8.80–18.44)	<0.001	12.65 (8.70–18.38)	<0.001
ASMQ	221/954	2.70 (1.90–3.82)	<0.001	2.62 (1.84–3.71)	<0.001
ASSP	49/121	4.18 (2.12–8.26)	<0.001	3.93 (1.97–7.84)	<0.001
DP	133/831	2.23 (1.53–3.26)	<0.001	2.16 (1.48–3.16)	<0.001
Q	18/144	2.79 (1.19–6.59)	0.02	2.43 (1.02–5.78)	0.05
QC	No data	Omitted			
Baseline fatigue					
Yes	545/1015	7.80 (6.37–9.54)	<0.001	8.07 (6.57–9.91)	<0.001
No	391/3231	Reference		Reference	
Age (years)	936/4246	1.02 (1.01–1.04)	0.004	1.03 (1.01–1.04)	0.001
Parity					
0	347/1871	Reference			
1	193/891	1.05 (0.83–1.33)	0.69		
≥2	372/1453	1.29 (1.05–1.58)	0.01		
EGA (weeks)	936/4243	1.00 (0.98–1.02)	0.96		
Weight (kg)	936/4233	1.00 (0.99–1.01)	0.63		
Parasitaemia (log10/µL)	936/4246	0.96 (0.86–1.07)	0.45		
Hyperparasitaemia					
Yes	21/48	1.66 (0.81–3.42)	0.17		
No	915/4198	Reference			
Fever					
Yes	134/371	1.13 (0.84–1.52)	0.41		
No	800/3856	Reference			
Haemoglobin (g/dL)	935/4212	0.96 (0.90–1.02)	0.19	0.93 (0.87–1.00)	0.04
Presence of gametocytes					
Yes	38/128	1.25 (0.78–2.00)	0.35		
No	871/4085	Reference			
Mixed infection					
Yes	10/27	0.87 (0.32–2.36)	0.78		
No	926/4219	Reference			
Malaria transmission intensity					
Low	267/702	2.33 (1.08–5.05)	0.03	2.11 (0.96–4.63)	0.06
Moderate	380/2518	Reference		Reference	
High	289/1026	4.04 (2.36–6.92)	<0.001	4.10 (2.39–7.04)	<0.001

* Adjusted by variables in the column. Intraclass correlation: 0.23. AAP: artesunate with atovaquone-proguanil, AC: artesunate with clindamycin, AL: artemether-lumefantrine, AS: artesunate monotherapy, ASAQ: artesunate-amodiaquine, ASMQ: artesunate-mefloquine, ASSP: artesunate-sulfadoxine-pyrimethamine, CI: confidence interval, DP: dihydroartemisinin-piperaquine, EGA: estimated gestational age, OR: odds ratio, Q: quinine monotherapy, QC: quinine with clindamycin.

Appendix Table 5-6. Univariable and multivariable logistic regression of the risk of developing headache on day 1–7

Baseline characteristic	Number of positive / assessed	Univariable		Multivariable*	
		OR (95% CI)	p-value	OR (95% CI)	p-value
Treatment					
AL	275/1229	Reference		Reference	
AAP	53/78	2.94 (1.05–8.25)	0.04	2.86 (1.00–8.19)	0.05
AC	2/9	Omitted			
AS	154/208	1.05 (0.53–2.08)	0.88	1.03 (0.52–2.02)	0.94
ASAQ	137/853	1.56 (1.14–2.13)	0.01	1.56 (1.14–2.14)	0.01
ASMQ	199/1003	1.21 (0.92–1.60)	0.17	1.21 (0.92–1.60)	0.17
ASSP	16/121	1.13 (0.50–2.54)	0.77	1.13 (0.50–2.57)	0.76
DP	161/831	1.22 (0.91–1.64)	0.17	1.24 (0.92–1.66)	0.16
Q	36/228	0.71 (0.37–1.37)	0.31	0.71 (0.36–1.38)	0.31
QC	41/63	1.25 (0.46–3.42)	0.67	1.21 (0.44–3.32)	0.71
Baseline headache					
Yes	793/1818	5.56 (4.64–6.66)	<0.001	5.56 (4.64–6.66)	<0.001
No	278/2802	Reference		Reference	
Age (years)	1074/4623	1.01 (0.99–1.02)	0.25		
Parity					
0	398/2001	Reference			
1	220/974	1.07 (0.86–1.34)	0.55		
≥2	439/1615	1.22 (1.01–1.48)	0.04		
EGA (weeks)	1074/4620	1.00 (0.99–1.02)	0.86		
Weight (kg)	1072/4606	1.00 (0.99–1.01)	0.50		
Parasitaemia (log10/µL)	1074/4623	1.00 (0.90–1.11)	0.98		
Hyperparasitaemia					
Yes	26/62	0.92 (0.48–1.74)	0.79		
No	1048/4561	Reference			
Fever					
Yes	172/435	1.23 (0.93–1.64)	0.14		
No	893/4139	Reference			
Haemoglobin (g/dL)	1072/4584	0.98 (0.92–1.04)	0.44		
Presence of gametocytes					
Yes	44/165	0.69 (0.43–1.10)	0.12		
No	1005/4422	Reference			
Mixed infection					
Yes	23/34	2.12 (0.76–5.92)	0.15		
No	1051/4589	Reference			
Malaria transmission intensity					
Low	464/1048	1.53 (0.67–3.52)	0.32	1.53 (0.67–3.52)	0.32
Moderate	427/2519	Reference		Reference	
High	183/1056	2.82 (1.59–5.00)	<0.001	2.82 (1.59–5.00)	<0.001

* Adjusted by variables in the column. Intraclass correlation: 0.29. AAP: artesunate with atovaquone-proguanil, AC: artesunate with clindamycin, AL: artemether-lumefantrine, AS: artesunate monotherapy, ASAQ: artesunate-amodiaquine, ASMQ: artesunate-mefloquine, ASSP: artesunate-sulfadoxine-pyrimethamine, CI: confidence interval, DP: dihydroartemisinin-piperaquine, EGA: estimated gestational age, OR: odds ratio, Q: quinine monotherapy, QC: quinine with clindamycin.

Appendix Table 5-7. Univariable and multivariable logistic regression of the risk of developing musculoskeletal pain on day 1–7

Baseline characteristic	Number of positive / assessed	Univariable		Multivariable*	
		OR (95% CI)	p-value	OR (95% CI)	p-value
Treatment					
AL	139/1184	Reference		Reference	
AAP	30/72	2.41 (0.72–8.04)	0.15	1.77 (0.56–5.62)	0.33
AC	1/9	Omitted			
AS	124/203	1.13 (0.62–2.08)	0.69	1.06 (0.58–1.94)	0.85
ASAQ	109/827	2.80 (1.87–4.20)	<0.001	2.83 (1.88–4.24)	<0.001
ASMQ	96/883	1.31 (0.89–1.93)	0.17	1.26 (0.85–1.86)	0.25
ASSP	No data	Omitted			
DP	85/831	1.14 (0.76–1.71)	0.53	1.13 (0.75–1.69)	0.56
Q	26/228	2.43 (0.94–6.29)	0.07	1.87 (0.73–4.77)	0.19
QC	27/62	0.73 (0.27–1.94)	0.52	0.71 (0.26–1.88)	0.49
Baseline musculoskeletal pain					
Yes	440/1027	8.35 (6.66–10.46)	<0.001	7.76 (6.17–9.76)	<0.001
No	193/3268	Reference		Reference	
Age (years)	637/4299	1.02 (1.00–1.04)	0.02		
Parity					
0	201/1843	Reference		Reference	
1	126/894	1.10 (0.82–1.47)	0.52	1.08 (0.80–1.45)	0.61
≥2	310/1555	1.47 (1.16–1.87)	0.002	1.45 (1.14–1.85)	0.003
EGA (weeks)	637/4296	1.02 (1.01–1.04)	0.01	1.02 (1.00–1.04)	0.03
Weight (kg)	636/4282	1.00 (0.99–1.02)	0.53		
Parasitaemia (log10/µL)	637/4299	1.06 (0.94–1.21)	0.34		
Hyperparasitaemia					
Yes	17/49	1.46 (0.64–3.31)	0.37		
No	620/4250	Reference			
Fever					
Yes	116/350	1.57 (1.11–2.22)	0.01	1.58 (1.11–2.24)	0.01
No	521/3905	Reference		Reference	
Haemoglobin (g/dL)	637/4262	0.99 (0.92–1.06)	0.74		
Presence of gametocytes					
Yes	29/154	0.81 (0.46–1.42)	0.47		
No	599/4134	Reference			
Mixed infection					
Yes	18/34	1.55 (0.57–4.21)	0.39		
No	619/4265	Reference			
Malaria transmission intensity					
Low	306/768	4.04 (1.39–11.73)	0.01	3.39 (1.19–9.64)	0.02
Moderate	207/2475	Reference		Reference	
High	124/1056	2.56 (1.47–4.46)	0.001	2.68 (1.53–4.69)	0.001

* Adjusted by variables in the column. Intraclass correlation: 0.26. AAP: artesunate with atovaquone-proguanil, AC: artesunate with clindamycin, AL: artemether-lumefantrine, AS: artesunate monotherapy, ASAQ: artesunate-amodiaquine, ASMQ: artesunate-mefloquine, ASSP: artesunate-sulfadoxine-pyrimethamine, CI: confidence interval, DP: dihydroartemisinin-piperaquine, EGA: estimated gestational age, OR: odds ratio, Q: quinine monotherapy, QC: quinine with clindamycin.

Appendix Table 5-8. Univariable and multivariable logistic regression of the risk of developing nausea on day 1–7

Baseline characteristic	Number of positive / assessed	Univariable		Multivariable*	
		OR (95% CI)	p-value	OR (95% CI)	p-value
Treatment					
AL	75/1225	Reference		Reference	
AAP	29/72	6.39 (2.27–18.00)	<0.001	4.70 (1.83–12.07)	0.001
AC	1/10	Omitted			
AS	79/203	1.15 (0.64–2.06)	0.63	1.23 (0.69–2.17)	0.48
ASAQ	112/827	6.56 (3.93–10.94)	<0.001	6.04 (3.68–9.91)	<0.001
ASMQ	208/1033	6.22 (3.86–10.05)	<0.001	5.68 (3.58–9.00)	<0.001
ASSP	10/121	1.54 (0.60–3.96)	0.37	1.30 (0.51–3.35)	0.59
DP	61/830	3.14 (1.82–5.41)	<0.001	2.88 (1.70–4.88)	<0.001
Q	76/227	8.35 (4.16–16.77)	<0.001	7.29 (3.78–14.08)	<0.001
QC	29/61	1.87 (0.70–4.98)	0.21	2.07 (0.80–5.39)	0.14
Baseline nausea					
Yes	307/599	7.36 (5.83–9.28)	<0.001	7.60 (6.02–9.61)	<0.001
No	369/3987	Reference		Reference	
Age (years)	680/4609	1.02 (1.01–1.04)	0.004	1.02 (1.01–1.04)	0.01
Parity					
0	245/2005	Reference			
1	152/977	1.04 (0.80–1.34)	0.78		
≥2	281/1620	1.18 (0.94–1.47)	0.14		
EGA (weeks)	680/4606	0.99 (0.97–1.00)	0.11		
Weight (kg)	680/4592	0.99 (0.98–1.01)	0.31		
Parasitaemia (log10/µL)	680/4609	1.00 (0.89–1.12)	0.99		
Hyperparasitaemia					
Yes	12/59	0.93 (0.42–2.03)	0.85		
No	668/4550	Reference			
Fever					
Yes	115/418	1.24 (0.92–1.67)	0.15		
No	563/4141	Reference			
Haemoglobin (g/dL)	675/4568	0.99 (0.92–1.06)	0.69		
Presence of gametocytes					
Yes	34/166	0.77 (0.48–1.23)	0.28		
No	644/4416	Reference			
Mixed infection					
Yes	10/33	1.01 (0.40–2.54)	0.99		
No	670/4576	Reference			
Malaria transmission intensity					
Low	338/1015	3.98 (2.04–7.78)	<0.001	3.95 (2.04–7.66)	<0.001
Moderate	188/2538	Reference		Reference	
High	154/1056	2.82 (1.59–5.02)	<0.001	2.76 (1.56–4.91)	0.001

* Adjusted by variables in the column. Intraclass correlation: 0.13. AAP: artesunate with atovaquone-proguanil, AC: artesunate with clindamycin, AL: artemether-lumefantrine, AS: artesunate monotherapy, ASAQ: artesunate-amodiaquine, ASMQ: artesunate-mefloquine, ASSP: artesunate-sulfadoxine-pyrimethamine, CI: confidence interval, DP: dihydroartemisinin-piperaquine, EGA: estimated gestational age, OR: odds ratio, Q: quinine monotherapy, QC: quinine with clindamycin.

Appendix Table 5-9. Univariable and multivariable logistic regression of the risk of developing tinnitus on day 1–7

Baseline characteristic	Number of positive / assessed	Univariable		Multivariable*	
		OR (95% CI)	p-value	OR (95% CI)	p-value
Treatment					
AL	41/1150	Reference		Reference	
AAP	26/75	5.05 (0.94–27.26)	0.06	6.29 (1.33–29.86)	0.02
AC	1/10	Omitted			
AS	76/205	1.64 (0.77–3.49)	0.20	1.73 (0.81–3.71)	0.16
ASAQ	12/827	5.09 (1.04–24.89)	0.04	4.24 (0.89–20.29)	0.07
ASMQ	37/1007	7.06 (1.72–28.99)	0.01	6.22 (1.56–24.82)	0.01
ASSP	4/121	2.05 (0.26–15.96)	0.49	1.67 (0.22–12.79)	0.62
DP	15/830	6.20 (1.23–31.19)	0.03	5.75 (1.19–27.77)	0.03
Q	168/228	211.99 (63.05–712.78)	<0.001	249.84 (80.90–771.56)	<0.001
QC	54/63	63.56 (17.33–233.10)	<0.001	71.91 (19.45–265.86)	<0.001
Baseline tinnitus					
Yes	182/287	48.00 (29.52–78.07)	<0.001	50.95 (31.13–83.40)	<0.001
No	250/4227	Reference		Reference	
Age (years)	434/4516	1.02 (0.99–1.04)	0.26		
Parity					
0	145/1956	Reference			
1	103/960	1.37 (0.88–2.14)	0.17		
≥2	186/1595	1.23 (0.83–1.83)	0.31		
EGA (weeks)	434/4513	1.00 (0.97–1.03)	0.94		
Weight (kg)	434/4499	0.99 (0.97–1.02)	0.52		
Parasitaemia (log10/µL)	434/4516	1.06 (0.88–1.28)	0.51		
Hyperparasitaemia					
Yes	13/55	0.52 (0.18–1.47)	0.22		
No	421/4461	Reference			
Fever					
Yes	95/409	0.97 (0.61–1.54)	0.90		
No	339/4092	Reference			
Haemoglobin (g/dL)	430/4476	1.07 (0.96–1.21)	0.23		
Presence of gametocytes					
Yes	47/159	1.75 (0.92–3.34)	0.09		
No	384/4350	Reference			
Mixed infection					
Yes	11/34	1.04 (0.31–3.49)	0.94		
No	423/4482	Reference			
Malaria transmission intensity					
Low	356/1018	4.26 (1.98–9.17)	<0.001	4.26 (1.98–9.17)	<0.001
Moderate	54/2472	Reference		Reference	
High	24/1026	5.90 (1.73–20.08)	0.005	5.90 (1.73–20.08)	0.005

* Adjusted by variables in the column. Intraclass correlation: 0.42. AAP: artesunate with atovaquone-proguanil, AC: artesunate with clindamycin, AL: artemether-lumefantrine, AS: artesunate monotherapy, ASAQ: artesunate-amodiaquine, ASMQ: artesunate-mefloquine, ASSP: artesunate-sulfadoxine-pyrimethamine, CI: confidence interval, DP: dihydroartemisinin-piperaquine, EGA: estimated gestational age, OR: odds ratio, Q: quinine monotherapy, QC: quinine with clindamycin.

Appendix Table 5-10. Univariable and multivariable logistic regression of the risk of developing vomiting on day 1–7

Baseline characteristic	Number of positive / assessed	Univariable		Multivariable*	
		OR (95% CI)	p-value	OR (95% CI)	p-value
Treatment					
AL	33/1223	Reference		Reference	
AAP	8/70	5.12 (1.77–14.79)	0.003	4.96 (1.66–14.80)	0.004
AC	0/9	Omitted			
AS	30/200	2.08 (1.03–4.19)	0.04	2.07 (1.03–4.17)	0.04
ASAQ	172/853	11.38 (6.95–18.62)	<0.001	11.56 (7.04–19.00)	<0.001
ASMQ	207/1025	10.15 (6.28–16.41)	<0.001	10.34 (6.38–16.76)	<0.001
ASSP	5/121	1.54 (0.50–4.76)	0.45	1.54 (0.50–4.75)	0.45
DP	70/830	4.09 (2.41–6.96)	<0.001	4.24 (2.49–7.21)	<0.001
Q	40/227	9.85 (4.88–19.88)	<0.001	9.61 (4.70–19.63)	<0.001
QC	16/60	9.21 (3.20–26.55)	<0.001	8.73 (3.00–25.37)	<0.001
Baseline vomiting					
Yes	157/351	6.61 (5.05–8.64)	<0.001	6.46 (4.93–8.46)	<0.001
No	421/4245	Reference		Reference	
Age (years)	581/4618	1.01 (0.99–1.02)	0.41		
Parity					
0	232/2001	Reference			
1	123/972	0.95 (0.74–1.23)	0.71		
≥2	211/1612	1.05 (0.84–1.31)	0.65		
EGA (weeks)	581/4615	0.98 (0.96–1.00)	0.03	0.98 (0.96–1.00)	0.03
Weight (kg)	581/4601	1.00 (0.98–1.01)	0.41		
Parasitaemia (log10/µL)	581/4618	0.99 (0.88–1.11)	0.89		
Hyperparasitaemia					
Yes	7/59	0.72 (0.29–1.80)	0.48		
No	574/4559	Reference			
Fever					
Yes	78/429	1.21 (0.89–1.65)	0.23		
No	502/4140	Reference			
Haemoglobin (g/dL)	577/4578	1.05 (0.98–1.12)	0.19		
Presence of gametocytes					
Yes	21/163	1.01 (0.61–1.68)	0.96		
No	543/4402	Reference			
Mixed infection					
Yes	3/32	0.73 (0.20–2.66)	0.64		
No	578/4586	Reference			
Malaria transmission intensity					
Low	157/1024	1.46 (0.86–2.49)	0.16	1.48 (0.87–2.54)	0.15
Moderate	254/2538	Reference		Reference	
High	170/1056	1.80 (1.09–2.96)	0.02	1.82 (1.10–3.01)	0.02

* Adjusted by variables in the column. Intraclass correlation: 0.08. AAP: artesunate with atovaquone-proguanil, AC: artesunate with clindamycin, AL: artemether-lumefantrine, AS: artesunate monotherapy, ASAQ: artesunate-amodiaquine, ASMQ: artesunate-mefloquine, ASSP: artesunate-sulfadoxine-pyrimethamine, CI: confidence interval, DP: dihydroartemisinin-piperaquine, EGA: estimated gestational age, OR: odds ratio, Q: quinine monotherapy, QC: quinine with clindamycin.

Reference for appendix

1. McGready R, Brockman A, Cho T, et al. Randomized comparison of mefloquine-artesunate versus quinine in the treatment of multidrug-resistant falciparum malaria in pregnancy. *Trans R Soc Trop Med Hyg* 2000; **94**(6): 689-93.
2. McGready R, Cho T, Samuel, et al. Randomized comparison of quinine-clindamycin versus artesunate in the treatment of falciparum malaria in pregnancy. *Trans R Soc Trop Med Hyg* 2001; **95**(6): 651-6.
3. McGready R, Ashley EA, Moo E, et al. A randomized comparison of artesunate-atovaquone-proguanil versus quinine in treatment for uncomplicated falciparum malaria during pregnancy. *J Infect Dis* 2005; **192**(5): 846-53.
4. Kalilani L, Mofolo I, Chaponda M, et al. A randomized controlled pilot trial of azithromycin or artesunate added to sulfadoxine-pyrimethamine as treatment for malaria in pregnant women. *PLoS One* 2007; **2**(11): e1166.
5. McGready R, Tan SO, Ashley EA, et al. A randomised controlled trial of artemether-lumefantrine versus artesunate for uncomplicated Plasmodium falciparum treatment in pregnancy. *PLoS Med* 2008; **5**(12): e253.
6. Piola P, Nabasumba C, Turyakira E, et al. Efficacy and safety of artemether-lumefantrine compared with quinine in pregnant women with uncomplicated Plasmodium falciparum malaria: an open-label, randomised, non-inferiority trial. *Lancet Infect Dis* 2010; **10**(11): 762-9.
7. PREGACT Study Group, Pekyi D, Ampromfi AA, et al. Four artemisinin-based treatments in African pregnant women with malaria. *N Engl J Med* 2016; **374**(10): 913-27.
8. Nambozi M, Kabuya J-BB, Hachizovu S, et al. Artemisinin-based combination therapy in pregnant women in Zambia: efficacy, safety and risk of recurrent malaria. *Malar J* 2017; **16**: 199.
9. Anvikar AR, Kuepfer I, Mishra V, et al. Efficacy of two artemisinin-based combinations for the treatment of malaria in pregnancy in India: a randomized controlled trial. *Malar J* 2018; **17**: 246.
10. McGready R, Stepniewska K, Edstein MD, et al. The pharmacokinetics of atovaquone and proguanil in pregnant women with acute falciparum malaria. *Eur J Clin Pharmacol* 2003; **59**(7): 545-52.
11. McGready R, Phyo AP, Rijken MJ, et al. Artesunate/dihydroartemisinin pharmacokinetics in acute falciparum malaria in pregnancy: absorption, bioavailability, disposition and disease effects. *Br J Clin Pharmacol* 2012; **73**(3): 467-77.
12. Rijken MJ, McGready R, Phyo AP, et al. Pharmacokinetics of dihydroartemisinin and piperaquine in pregnant and nonpregnant women with uncomplicated falciparum malaria. *Antimicrob Agents Chemother* 2011; **55**(12): 5500-6.
13. Valea I, Tinto H, Traore-Coulibaly M, et al. Pharmacokinetics of co-formulated mefloquine and artesunate in pregnant and non-pregnant women with uncomplicated Plasmodium falciparum infection in Burkina Faso. *J Antimicrob Chemother* 2014; **69**(9): 2499-507.
14. Juma EA, Ogutu BR, Oloo F, Barwa T, Aman R. Pharmacokinetics of artemether-lumefantrine in pregnant and non-pregnant women with uncomplicated Plasmodium falciparum malaria in western Kenya. *Am J Trop Med Hyg* 2014; **91**(5 Supplement1): 587.
15. Mosha D, Guidi M, Mwingira F, et al. Population pharmacokinetics and clinical response for artemether-lumefantrine in pregnant and nonpregnant women with uncomplicated Plasmodium falciparum malaria in Tanzania. *Antimicrob Agents Chemother* 2014; **58**(8): 4583-92.
16. Nyunt MM, Nguyen VK, Kajubi R, et al. Artemether-lumefantrine pharmacokinetics and clinical response are minimally altered in pregnant Ugandan women treated for uncomplicated falciparum malaria. *Antimicrob Agents Chemother* 2016; **60**(3): 1274-82.
17. Ndiaye JL, Ndiaye A, Faye B, et al. Open-label in vivo drug study to evaluate the safety and efficacy of artesunate plus amodiaquine combination in pregnant women with uncomplicated P. falciparum malaria in Senegal. *Trop Med Int Health* 2011; **16**(Supplement 1): 140.
18. McGready R, Keo NK, Villegas L, White NJ, Looareesuwan S, Nosten F. Artesunate-atovaquone-proguanil rescue treatment of multidrug-resistant Plasmodium falciparum malaria in pregnancy: a preliminary report. *Trans R Soc Trop Med Hyg* 2003; **97**(5): 592-4.
19. Rijken MJ, McGready R, Boel ME, et al. Dihydroartemisinin-piperaquine rescue treatment of multidrug-resistant Plasmodium falciparum malaria in pregnancy: a preliminary report. *Am J Trop Med Hyg* 2008; **78**(4): 543-5.
20. Cohee LM, Kalilani-Phiri L, Mawindo P, et al. Parasite dynamics in the peripheral blood and the placenta during pregnancy-associated malaria infection. *Malar J* 2016; **15**: 483.
21. Kalilani-Phiri L, Thesing PC, Nyirenda OM, et al. Timing of malaria infection during pregnancy has characteristic maternal, infant and placental outcomes. *PLoS One* 2013; **8**(9): e74643.

22. McGready R. Randomised Trial of 3 Artemisinin Combination Therapy for Malaria in Pregnancy (NCT01054248). 2010. https://clinicaltrials.gov/ct2/show/NCT01054248.
23. Onyamboko MA, Fanello CI, Turner G, et al. Comparison of two regimens of artemether-lumefantrine for the treatment of uncomplicated Plasmodium falciparum malaria in pregnant women in the Democratic Republic of Congo. *Am J Trop Med Hyg* 2015; **93**(4 Supplement): 6.
24. Chico RM, Chandramohan D, Greenwood B, Mosha F. Cardio-Safety of Dihydroartemisinin-Piperaquine and Pharmacokinetics of Piperaquine Amongst Pregnant Women in Tanzania (NCT02909712). 2016. https://clinicaltrials.gov/ct2/show/NCT02909712.
25. Adam I, Ibrahim MH, A/elbasit IA, Elbashir MI. Low-dose quinine for treatment of chloroquine-resistant falciparum malaria in Sudanese pregnant women. *East Mediterr Health J* 2004; **10**(4/5): 554-9.
26. Adam I, Elwasila E, Mohammed Ali DA, Elansari E, Elbashir MI. Artemether in the treatment of falciparum malaria during pregnancy in eastern Sudan. *Trans R Soc Trop Med Hyg* 2004; **98**(9): 509-13.
27. Adam I, Ali DM, Abdalla MA. Artesunate plus sulfadoxine-pyrimethamine in the treatment of uncomplicated Plasmodium falciparum malaria during pregnancy in eastern Sudan. *Trans R Soc Trop Med Hyg* 2006; **100**(7): 632-5.
28. Adam I, Tarning J, Lindegardh N, Mahgoub H, McGready R, Nosten F. Pharmacokinetics of piperaquine in pregnant women in Sudan with uncomplicated Plasmodium falciparum malaria. *Am J Trop Med Hyg* 2012; **87**(1): 35-40.
29. Bounyasong S. Randomized trial of artesunate and mefloquine in comparison with quinine sulfate to treat P. falciparum malaria pregnant women. *J Med Assoc Thai* 2001; **84**(9): 1289-99.
30. Carmona-Fonseca J, Agudelo-García OM, Arango-Flórez E. [Therapeutic efficacy and adverse events of treatments for vivax and falciparum malaria in pregnant women in the regions of Uraba and Alto San Jorge, Colombia, 2008-2011] (in Spanish). *Rev Colomb Obstet Ginecol* 2013; **64**(1): 27-37.
31. Kaye DK, Nshemerirwe R, Mutyaba TS, Ndeezi G. A randomized clinical trial comparing safety, clinical and parasitological response to artemether-lumefantrine and chlorproguanil-dapsone in treatment of uncomplicated malaria in pregnancy in Mulago hospital, Uganda. *J Infect Dev Ctries* 2008; **2**(2): 135-9.
32. Mutabingwa TK, Muze K, Ord R, et al. Randomized trial of artesunate+amodiaquine, sulfadoxine-pyrimethamine+amodiaquine, chlorproguanal-dapsone and SP for malaria in pregnancy in Tanzania. *PLoS One* 2009; **4**(4): e5138.
33. Mutagonda RF, Kamuhabwa AAR, Minzi OMS, et al. Effect of pharmacogenetics on plasma lumefantrine pharmacokinetics and malaria treatment outcome in pregnant women. *Malar J* 2017; **16**: 267.
34. Mutagonda RF, Kamuhabwa AAR, Minzi OMS, Massawe SN, Maganda BA, Aklillu E. Malaria prevalence, severity and treatment outcome in relation to day 7 lumefantrine plasma concentration in pregnant women. *Malar J* 2016; **15**: 278.
